# Hypoxia Induced Changes of Exosome Cargo and Subsequent Biological Effects

**DOI:** 10.3389/fimmu.2022.824188

**Published:** 2022-04-04

**Authors:** Hongxia Jiang, Hanqiu Zhao, Mengzhe Zhang, Yuanzhou He, Xiaochen Li, Yongjian Xu, Xiansheng Liu

**Affiliations:** ^1^ Department of Pulmonary and Critical Care Medicine, Tongji Hospital, Tongji Medical College, Huazhong University of Science and Technology, Wuhan, China; ^2^ Key Laboratory of Pulmonary Diseases, National Ministry of Health of The People’s Republic of China, Wuhan, China

**Keywords:** hypoxia, exosome, cargo, tumor, changes

## Abstract

Exosomes are small extracellular vesicles that are secreted by almost all types of cells and exist in almost all extracellular spaces. As an important mediator of intercellular communication, exosomes encapsulate the miRNA, lncRNA, cirRNA, mRNA, cytokine, enzyme, lipid, and other components from the cytoplasm into its closed single membrane structure and transfer them to recipient units in an autocrine, paracrine, or endocrine manner. Hypoxia is a state of low oxygen tension and is involved in many pathological processes. Hypoxia influences the size, quantity, and expression of exosome cargos. Exosomes derived from hypoxic tumor cells transfer genetics, proteins, and lipids to the recipient units to exert pleiotropic effects. Different donor cells produce different cargo contents, target different recipient units and lead to different biological effects. Hypoxic exosomes derived from tumor cells uptaken by normoxic tumor cells lead to promoted proliferation, migration, and invasion; uptaken by extracellular space or liver lead to promoted metastasis; uptaken by endothelial cells lead to promoted angiogenesis; uptaken by immune cells lead to promoted macrophage polarization and changed tumor immune microenvironment. In addition to various types of tumors, hypoxic exosomes also participate in the development of diseases in the cardiovascular system, neuron system, respiratory system, hematology system, endocrine system, urinary system, reproduction system, and skeletomuscular system. Understanding the special characteristics of hypoxic exosomes provide new insight into elaborating the pathogenesis of hypoxia related disease. This review summarizes hypoxia induced cargo changes and the biological effects of hypoxic exosomes in tumors and non-malignant diseases in different systems.

## Introduction

Hypoxia, a state of inadequate oxygen supply, is a common feature of many different diseases, like solid tumor (1), hypoxia induced pulmonary arterial hypertension (2), sleep-disordered breathing (3), hypoxic kidney injury (4), placental hypoxia (5) and so on. The main molecular mechanism to sense oxygen stress is Hypoxia Induced Factors (HIFs), which functions as a master regulator of oxygen homeostasis in all metazoan species (6). HIFs are dimeric proteins composed of an oxygen-sensitive -subunit (HIF-1α, HIF-2α, or HIF-3α) and a β-subunit (HIF-1β) (7). HIF-1α is ubiquitously expressed whereas HIF-2α and HIF-3α are selectively expressed in certain tissues (8). Under normoxia, HIF-α subunits are hydroxylated by prolyl hydroxylases (PHD1-3) and recognized and targeted for proteasomal degradation. The activity of PHD1-3 is oxygen dependent, so under hypoxia, the rate of HIF-α hydroxylation is suppressed (9), accumulated HIF-1α dimerizes with HIF-1β and binds to hypoxia-responsive elements(HREs) in promoters of target genes to promote a concerted transcriptional response (10).

Hundreds of genes are now known to respond directly or indirectly to hypoxia *via* HIFs (11). Human large-scale genomic sequencing projects have revealed that less than 2% of transcriptional output encodes for proteins, while the remaining genome encrypts different classes of non-coding RNAs, including miRNA, lncRNA, cirRNA, and so on (12, 13). Hypoxia upregulates or downregulates the expression of some key factors, including the non-coding RNAs, as well as mRNAs, proteins, and lipids in hypoxic cells and the hypoxic exosomes secreted by these cells. The upregulated or downregulated key factors were transferred to recipient cells or tissues through exosomes, leading to various biological effects involving angiogenesis, invasion, metastasis, and immune escape in tumor development (14), and other hypoxia involved disease mentioned above. Recently, myriads of research exploring hypoxia induced changes in exosomes loads and subsequent effects have emerged rapidly. This review tries to focus on the impact of hypoxia on exosomes secretion and cargo changes, and summarizing various biological results in various diseases.

## Biogenesis and Recipience of Exosomes

The exosome is one kind of extracellular vesicle. Extracellular vesicles are mainly classified into three types according to biogenesis. Namely, apoptotic bodies range from 500-2000 nm, microvesicles range from 200-2000 nm and exosomes range from 40-200 nm (15). The nomenclature of extracellular vesicles has not reached a broad agreement today (16–18), and in this review, we only discuss those studies clearly stated “exosome” in their elaboration. The formation of exosomes is the budding process of membrane organizations. Firstly, the inward budding of the cell membrane leads to the formation of the endosome (19). Endosome goes through different stages: early endosome, recycling endosome, and end endosome (20), during the maturation from early endosome to end endosome, further inward budding inside an intracellular endosome occurs and leads to the formation of multivesicle body, characterized by the presence of intraluminal vesicles (21–23). During the process of endosome inward budding, cytosolic contents, transmembrane, and peripheral proteins are incorporated into the invaginating membrane (24). The membrane of the multivesicle body fuses either with lysosome leading to the degradation of vesicles contents (25) or fuses with cell membrane leading to the release of vesicles contents, namely exosomes (26, 27). In contrast to inward budding, the outward budding process leads to the formation of microvesicles or apoptotic bodies (15).

**Figure 1:**
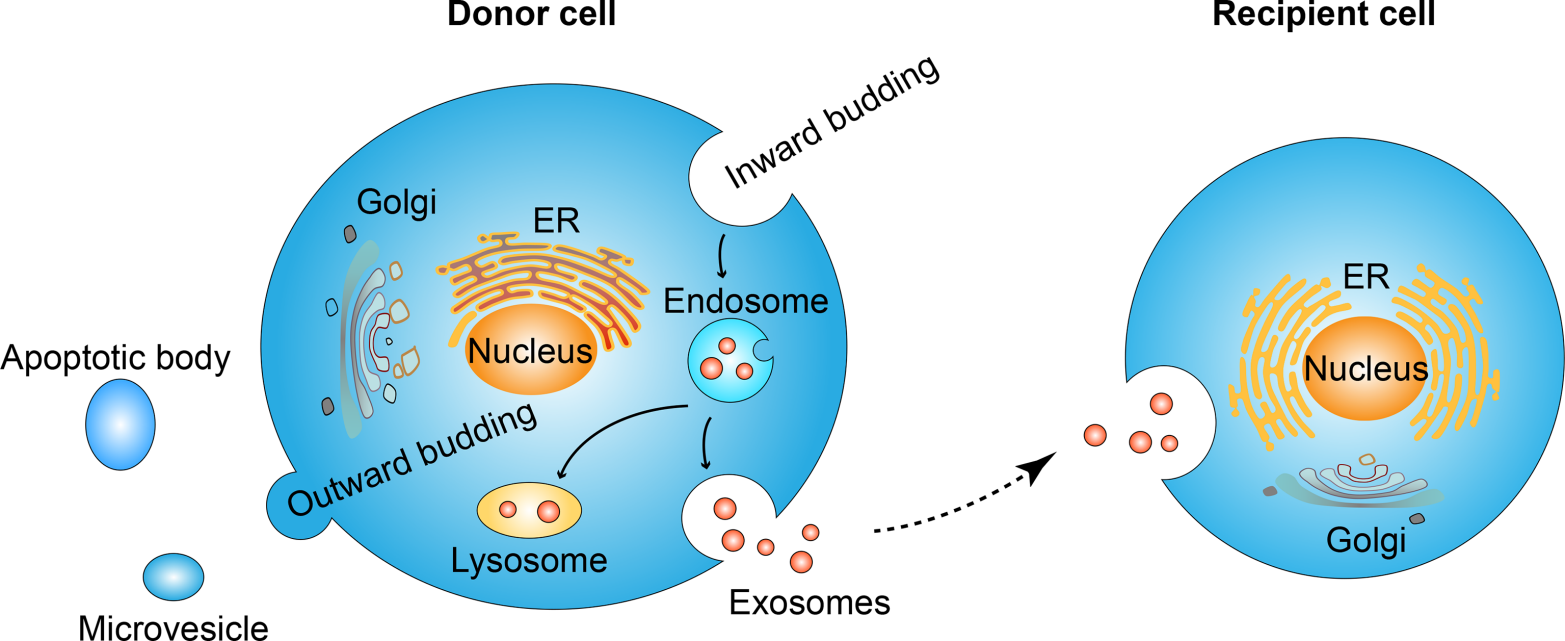
Exosome formation and uptaking process. Inward budding of cell membrane leads to the formation of the endosome. Further inward budding which encapsulates cell components leads to the formation of vesicles inside the endosome. The membrane of endosome fuse either with lysosome and result in the degradation of endosome contents, or fuse with cell membrane and lead to the release of its vesicles, namely exosomes. Secreted exosomes will be uptaken by recipient cells and the internalized contents are thus transferred in an autocrine, paracrine, or endocrine manner. In contrast to the inward budding process, the out ward budding of the membrane leads to the formation of microvesicles or apoptotic bodies.

Extracellular vesicles were first found during the maturation of sheep reticulocytes in 1983 (28). In the beginning, people thought it was just a kind of cell debris for the disposal of cell waste (22, 29). Later, researchers found that exosomes encapsulate RNAs, DNA, proteins, and lipids, and play a significant role both in cell to cell and cell to its milieu communication by transferring its encapsulated contents, both locally and distally (30, 31) and these contents are not randomized distributed but a specific subset from endosomes, the plasm membrane and the cytosol (32), suggesting an endosomal sorting complex required for transport (ESCRT) during exosome formation. The ESCRT dependent and independent manner of sorting mechanism are described in detail by Guillaume van Niel (33). As for the uptake mechanisms of exosomes by recipient cells, at least five mechanisms are involved, including clathrin-dependent, micropinocytosis, lipid-raft, membrane fusion, and caveolin-dependent endocytosis (34). In another word, exosomes sort specific contents to load and uptaken by specific recipient cells to exert their function (**Figure 1**). In addition, exosomes are secreted by almost all kinds of cells (35, 36) in biological and pathological conditions. And exists in almost all body fluids like blood, urine, saliva, synovial fluid, bile, cerebrospinal fluid, bronchoalveolar fluid, nasal fluid, uterine fluid, amniotic fluid, breast milk, feces, seminal plasma (30). It has been reported that many factors, including hypoxia, the change in pH, temperature, oxidative stress, radiation, and shear stress can affect the secretion level and composition of exosomes (37). Here, we focus on the impact of hypoxia on the production and cargo changes of exosomes and subsequent biological effects, and we made a brief summary in **Table 1**.

**Table 1:** Summary of hypoxia induced changes of exosome cargos from different donor units to their corresponding target recipient units, and leading to different biological effects.

Related systems	Donor units	Hypoxia induced changes of exosome contents	Recipient units	Biological effects	Ref
Tumors	Glioma cells	Hypoxia-regulated mRNAs and proteins (matrix metalloproteinases, IL-8, PDGFs, caveolin 1, and lysyl oxidase) ↑	Endothelial cells, glioma cells	Promoted angiogenesis ex vivo and *in vitro*; increased autocrine, promigratory activation of GBM cells	(1)
	Glioblastoma cells (GBM)	Tissue factor ↑	Endothelial cells	Elicited an angiogenic phenotype	(38)
	Glioma tumor tissue	miR-10a ↑, miR-21 ↑	Myeloid-derived suppressor cells (MDSCs)	Induced MDSC expansion and activation	(39)
	CD133+ U87 glioblastoma cells	4 miRNAs ↑, 6 miRNAs ↓	Human brain microvascular endothelial cells (HBMEC) and U87 glioma cells	Increased cell viability	(40)
	Glioblastoma cells	Protein-lysine 6-oxidase (LOX) ↑, thrombospondin-1 (TSP1) ↑, vascular derived endothelial factor (VEGF) ↑ and a disintegrin and metalloproteinase with thrombospondin motifs 1 (ADAMTS1) ↑	Recipient glioma cells, endothelial progenitor cells (EPCs), neighboring normoxic tumor cells and possibly in surrounding stromal cells,	Induced differential gene expression in recipient glioma cells, increased various angiogenic, induced marked changes in the expression of genes	(41)
	Glioma tumor tissue	miR-210 ↑	Not mentioned	Served as a diagnostic, prognostic and hypoxic biomarker to reflect glioma status and hypoxic signatures.	(42)
	Glioma	Interleukin 6 (IL-6) ↑, miR-155-3p ↑	Macrophages, nude mice	Promoted glioma proliferation and migration *in vitro* and *in vivo*	(43)
	Hypoxic glioma	miR-1246 ↑, miR-10b-5p ↑	Normoxic glioma	Promoted migration and invasion	(44)
	Hypoxic Glioma Stem Cell	lnc01060 containing	Glioma	Promoted Progression	(45)
	Glioma	miRNA-199a-3p ↑	Peritumoral Neurons	Increased the ischemic injury of peritumoral neurons by inhibiting the mTOR pathway	(46)
	Glioblastoma cells	miR-182-5p ↑	Human umbilical vein endothelial cells	Promoted tumor angiogenesis, enhanced vascular permeability and tumor trans endothelial migration	(47)
	Glioma	miR-1246 ↑	M2 macrophage	Markedly induced M2 macrophage polarization, which subsequently promoted glioma proliferation, migration and invasion *in vitro* and *in vivo*	(48)
	Hypoxic glioma cells	MCT1 ↑, CD147 ↑	Neighboring cells	Promoted tumor progression	(49)
	Hypoxic glioblastoma cells	miR-301a	Normoxic glioma cells	Promote radiation resistance.	(50)
	hypoxic glioblastoma cells	VEGF-A ↑	None	Enhanced the permeability of blood-brain-barrier	(51)
	Ovarian cancer cells	Potent oncogenic proteins-STAT3 and FAS	Immortalized fallopian tube secretory epithelial cells (FT)	Pro-tumorigenic in mouse fallopian tubes, cisplatin efflux *via* exosomes was significantly increased	(52)
	Ovarian cancer cells	miR‐199a‐5p ↓	Endothelial cells	Suppressing tumor metastasis	(53)
	Epithelial ovarian cancer (EOC)	miRNA-940 ↑	Macrophages	Stimulating TAM polarization	(54)
	Endometrial cancer KEL cells	miRNA-21 ↑	Monocyte cell line THP-1	Promoted monocyte THP-1 cell transformation to M2-like polarization macrophages	(55)
	Epithelial ovarian cancer cells	miR-21–3p ↑, miR-125 b-5p ↑, miR-181 d-5p ↑	Macrophages	Induced the polarization of M2 macrophages, which promoted EOC cell proliferation and migration in a feedback loop	(56)
	Breast cancer cell lines	Transforming growth factor-β, TGF-β	T cells	Suppressing T cell proliferation	(57)
	Breast cancer cells	MiR210 ↑	Surrounding tissue in the tumor microenvironment	Promoted their own survival and invasion	(58)
	Breast cancer cells	miR-210 ↑	Neighboring cells	Promoted angiogenesis	(59)
	Breast cancer cells	lncRNA SNHG1 ↑	HUVECs	promote the proliferation, migration and angiogenesis of HUVECs	(60)
	Breast cancer associated fibroblasts	GPR64 ↑	Breast cancer cells	Enhanced cancer cell invasive abilities	(61)
	Bone marrow-derived human mesenchymal stem cells	miR-let-7f ↑	4T1 cells	Attenuated proliferation and invasion	(62)
	Natural killer (NK) cells: NK92 or NK92-hIL-15 cells	NK cells-specifically FasL ↑, perforin ↑, and granzyme B ↑	MCF-7 breast cancer and A2780 ovarian cancer cells	Significantly increased cytotoxicity, enhanced inhibition of cell proliferation	(63)
	Human prostate cancer cell line PC3 cells	MMP2 ↑, MMP9 ↑, extracellular matrix proteins (fibronectin and collagen) ↑	Nude mice	Promoted matrix metalloproteinases (MMPs) activity in several putative metastatic sites	(64)
	Bladder cancer cells	lncRNA-UCA1 ↑	Bladder cancer UMUC2 cells with low expression of lncRNA-UCA1	Promoted cell proliferation, migration and invasion. promote tumor growth and progression through epithelial-mesenchymal transition, *in vitro* and *in vivo*	(65)
	LNCaP and PC3 cells	MMP-2 ↑, MMP-9 ↑, cytokines and signaling molecules (TGF-β2, TNF1α, IL6, TSG101, Akt, ILK1, β-catenin) ↑	LNCaP and PC3 cells	Increased the invasiveness and motility of naïve LNCaP and PC3 cells, respectively. Promoted prostasphere formation by both LNCaP and PC3 cells, and enhanced α-SMA (a CAF biomarker) expression in prostate stromal cells	(66)
	Prostate cancer (PCa) cells	Lactic acid	None	Removed metabolic waste	(67)
	RCC cell lines	CA9	Human umbilical vein endothelial cells (HUVEC)	Promoted migration and tube formation, increased MMP2 expression, enhanced angiogenesis	(68)
	Hypoxic renal cell carcinoma cells	lncHILAR ↑	Normoxic renal cell carcinoma cells	Promoted RCC cell invasion and metastasis	(69)
	LNCaP cells	Top 11 miRNAs ↑, Top 9 miRNAs ↓	Not mentioned	Potential usefulness as a biomarker of hypoxia in PCa patients	(70)
	Hypoxic tumor-associated macrophages	miR-155-5p ↑	Clear cell renal cell carcinoma	Promoted renal cell carcinoma (RCC) progression	(71)
	Plasma	miR-92a-3p ↑, miR-709 ↑, miR-671-5p ↓, miR-882 ↓	Mouse TC1 and human adenocarcinoma cells lines	Significantly promoted TC1 malignant properties, enhanced proliferation and migration of human adenocarcinoma cells	(72)
	Lung cancer cells	miR-23a ↑	Endothelial cells	Enhanced angiogenesis, increased vascular permeability and cancer transendothelial migration	(73)
	Lung cancer cell lines	TGF-β and IL-10	HMEC-1	Promoting the migration of cancer cells	(74)
	Bone marrow-derived mesenchymal stem cells (BMSCs)	miR-193a-3p, miR-210-3p and miR-5100	Lung cancer cells	promoted cancer cell invasion and EMT	(75)
	Tumor tissue of lung adenocarcinoma (LUAD)	lncRNA-p21 ↑	Human umbilical vein endothelial cells (HUVECs)	Promoting tube formation and enhancing tumor cell adhesion to endothelial cells	(76)
	Hypoxic LUAD cells	miR-31-5p	Normoxic LUAD cells	Significantly enhanced the migration and invasion, contributing to tumor progression both *in vitro* and *in vivo*	(77)
	LUAD cell lines	circSETDB1	Normoxic LUAD cells.	Improved the migration, invasion, and proliferation	(78)
	A549 cells	miR101 ↓	THP-1 cells	Activated macrophages to induce inflammation in the tumor microenvironment	(79)
	A549 cells	HIF-1α/COX-2 ↑, miR-135b and miR-210 ↑	A549 cells	promoted the proliferation, migration, and angiogenesis of other A549 cells	(80)
	Hypoxic NSCLC cells	miR-21 ↑	Normoxic NSCLC cells	Facilitated normoxic cell resistance to cisplatin	(81)
	Cisplatin-resistant NSCLC cells	PKM2 ↑	NSCLC cells, cancer-associated fibroblasts (CAFs)	Elicted cisplatin resistance in sensitive NSCLC cells	(82)
	Non-Small-Cell Lung Cancer Cell	miR-582-3p ↑	Normoxic NSCLC cells	Promoted the proliferation, migration, and invasion of normoxic NSCLC cells	(83)
	A549 cells	Angiopoietin-like 4 (ANGPTL4)	A549 cells or human umbilical vein endothelial cells (HUVECs)	Promoted the proliferation, migration and invasion of A549 cells as well as the proliferation and angiogenesis of HUVECs.	(84)
	Pancreatic cancer cells	miR-301a-3p ↑	Activate macrophages to the M2 phenotype in a HIF-1α or HIF-2α-dependent manner	Facilitated the migration, invasion, and epithelial-mesenchymal transition of pancreatic cancer cells	(85)
	BSp73ASML cells	C4.4A- and α6β4-associated MMP14	Not mentioned	Promoted migration on LN111 and LN332	(86)
	Hypoxic pancreatic cancer cells	circZNF91 ↑	Normoxic pancreatic cancer cells	Enhanced gemcitabine resistance	(87)
	Pancreatic stellate cells (PSCs)	miR-4465 ↑, miR-616-3p ↑	Pancreatic cancer cells	Promoting PC cell proliferation, migration, and invasion	(88)
	Pancreatic cancer (PC) cells	lncRNA UCA1 ↑	HUVECs	Promoted angiogenesis and tumor growth through the miR-96-5p/AMOTL2/ERK1/2 axis	(89)
	Pancreatic ductal adenocarcinoma (PDAC) cells	miR-30b-5p ↑	HUVEC	Promoted tube formation and angiogenesis	(90)
	Pancreatic stellate cells (PSCs)	lncRNA UCA1 ↑	Pancreatic cancer cells	Promoted Gemcitabine resistance and tumorigenesis by regulating the EZH2/SOCS3 axi**s**	(91)
	Colorectal cancer (CRC) cells	Wnt4	Endothelial cells	Promoted angiogenesis through exosome-mediated Wnt/β-catenin signaling in endothelial cells	(92)
	Primary colorectal cancer (CRC)	miR-135a-5p ↑	liver	Initiated LATS2-YAP-MMP7 axis to promote the occurrences of CRC liver metastasis	(93)
	Hypoxic colorectal cancer cells	miR-210-3p ↑	Normoxic tumor cells	Elicited protumoral effects	(94)
	colorectal cancer	miR-361-3p ↑	colorectal cancer cells	Facilitated cell growth and suppressed cell apoptosis	(95)
	Hypoxic colorectal cancer cells	Circ-133 ↑	Normoxic colorectal cancer cells	Promoted cancer metastasis by acting on miR-133a/GEF-H1/RhoA axis	(96)
	Hypoxic colorectal cancer (CRC) cells	miR-410-3p ↑	Normoxic colorectal cancer (CRC) cells	Enhanced tumor progression	(97)
	Hypoxic colorectal cancer cells	Wnt4 ↑	Normoxic colorectal cancer cells	Promoted the migration and invasion abilities of normoxic CRC cells	(98)
	Hypoxic colorectal cancer cells	miR-1255b-5p ↓	Normoxic colorectal cancer cells	Enhanced epithelial-to-mesenchymal transition	(99)
	Colon cancer cells	Not mentioned	Colon cancer cells	Promoted self-growth	(100)
	Hypoxic colorectal cancer cell lines	miR-486-5p ↓, miR-181a-5p ↓, miR-30d-5p ↑	Not investigated	Retrieved as circulating markers of high-risk locally advanced rectal cancer	(101)
	Cancer-associated fibroblast	circEIF3K ↑	Human colorectal cancer cells HCT116 and SW620	Contributed to the proliferation, invasion and tube formation of recipient cells	(102)
	Granulocytic myeloid-derived suppressor cells	S100A9 ↑	Colorectal cancer cells	Promoted CRC cell stemness and growth	(103)
	Oesophageal squamous cell carcinoma cells	miR-340-5p ↑	Oesophageal squamous cell carcinoma cells	Alleviated radiation-induced apoptosis and accelerated DNA damage repair, leading to radioresistance	(104)
	ESCC Cells	hsa-circ-0048117↑	Macrophages	Promoted M2 polarization and M2 macrophages could enhance the ability of invasion and migration of tumor cells	(105)
	Nasopharyngeal carcinoma (NPC)	MMP-13	normoxic cells	Enhanced migration and invasiveness and induce microenvironment changes to promote NPC aggressiveness, enhancing the metastases of normoxic cells	(106)
	Adipocyte	miR-433-3p ↓	Nasopharyngeal carcinoma cells	Promoted proliferation, migration, and lipid accumulation	(107)
	Hypoxic hepatocellular carcinoma cells	miR-1273f ↑	Normoxic hepatocellular carcinoma cells	Enhanced the proliferation, migration, and invasiveness in addition to epithelial-to-mesenchymal transition (EMT)	(108)
	Hypoxic hepatocellular carcinoma	miR-155 ↑	HUVECs	Enhanced tube formation of HUVECs	(109)
	HepG2 cells	miR23a ↑	HUVECs	Induced angiogenesis.	(110)
	Hypoxic hepatocellular carcinoma Huh7 cells	Not mentioned	Normoxic hepatocellular carcinoma Huh7 cells	Promoted cell proliferation, migration and invasion	(111)
	hepatocellular carcinoma (HCC) cells	lncRNA HMMR-AS1 ↑	Macrophages	Promoted the M2 macrophages polarization and accelerated the progression of HCC	(112)
	Oral squamous cell carcinoma (OSCC)	miR-21 ↑	Normoxic cells	Promoted prometastatic, increased the migration and invasion of OSCC cells	(113)
	OSCC cell lines: Cal-27 and SCC-9	miR-21 ↑	Gammadelta T cells	Enhanced the suppressive effect of myeloid-derived suppressor cells (MDSCs) on gammadelta T cells	(114)
	Esophageal squamous cell carcinoma (ESCC) cells	Not mentioned	HUVECs, nude mice	Promoted proliferation, migration, invasion and tube formation of HUVECs. Enhanced the tumor growth and lung metastasis in nude mice models	(115)
	Gastric cancer cells	miR-301a-3p ↑	Gastric cancer cells	Facilitated GC proliferation, invasion, migration, and epithelial-mesenchymal transition	(116)
	Papillary thyroid cancer (PTC)	miR-181a ↑	Human umbilical vein endothelial cells (HUVECs)	Promoted proliferation and capillary-like network formation	(117)
	A431 carcinoma cells	Proteins	Not mentioned	Enhanced angiogenic and metastatic potential, modulate their microenvironment and facilitate angiogenesis and metastasis.	(118)
	Melanoma cell lines	miR-494-5p ↑, miR-4497 ↑, miR-513a-5p ↑, miR- 6087 ↑, miR-4454 ↑, miR-4299 ↑	Neighboring melanoma cells	Facilitated invasion and metastases.	(119)
	Melanoma cell lines: CRL-1424 and CRL-1675 cells	miRNAs were differently expressed in hypoxic exosomes	THP1 macrophages	Increased M1 markers (CXCL10 and IL6) in monocytes	(120)
	Mouse melanoma B16-F0 cells	CSF-1 ↑, CCL2 ↑, FTH ↑, FTL ↑, and TGF-beta ↑, miRNA let-7a ↑	Macrophages	Promoted M2-like polarization, enhanced oxidative phosphorylation of macrophages	(121)
	Endothelial cells	1,354 proteins (top changed): SEMG1 ↑, CO4A ↑, LOXL2 ↑, CO1A1 ↓, AN32E ↓, EPN1 ↓; 1,992 mRNAs(top changed): NDRG1 ↑, BNIP3 ↑, CIRBP ↓	Extracellular matrix	Cytoskeletal and extracellular matrix rearrangements	(122)
	Hypoxic Ewing’s sarcoma (EWS) cells	miR-210 ↑	Ewing’s sarcoma (EWS) cells	Promoted sphere formation, a stem-like phenotype	(123)
	Hypoxic multiple myeloma cells	miR-1305 ↑	Macrophages	Promoted tumor development	(124)
	Hypoxia-resistant multiple myeloma cells	miR-135b ↑	Endothelial cells	Suppressing the target factor-inhibiting hypoxia-inducible factor 1 (FIH-1), enhance endothelial tube formation	(125)
Cardiovascular system	Cardiomyoblast cells (H9c2)	miR-21-5p, miR-378-3p, miR-152-3p, and let-7i-5p	None	Mitigated hypoxia-induced H9c2 cells apoptosis	(126)
	Mouse bone marrow-derived MSCs	miR-210 ↑, neutral sphingomyelinase 2 (nSMase2) ↑	Infarcted heart	Significantly higher survival, smaller scar size and better cardiac functions recovery; increased vascular density, lower cardiomyocytes (CMs) apoptosis; reduced fibrosis and increased recruitment of cardiac progenitor cells in the infarcted heart	(127)
	Endothelial colony-forming cells (ECFCs)	miR-10b-5p ↓, neutral sphingomyelinase 2 (N-SMase2) ↓	Cardiac fibroblast	Anti-fibrotic effects of hypoxic exosomes were abolished	(128)
	Endothelial cells	6 miRNAs (including has-mir-383-3p) differently expressed	Endothelial cells	Promoted increased permeability and dysfunction of endothelial cells *in vitro*	(129)
	Mesenchymal stem cells (MSCs)	lncRNA-UCA1 ↑	Rats with myocardial infarction	Played a cardioprotective role	(130)
	Cardiomyocytes	circHIPK3 ↑	Cardiac microvascular endothelial cells	Inhibiting miR-29a activity, leading to increased IGF-1 expression, exhibiting CMVECs protection	(131)
	Cardiomyocytes	circHIPK3 ↑	Cardiac endothelial cells, mice with myocardial infarction	Promoted cardiac endothelial cell migration, proliferation, and tube formation *in vitro*; effectively reduced the infarct area and promote angiogenesis in the border surrounding the infarcted area	(132)
	Cardiomyocytes	miR-30a ↑	Cardiomyocytes	Inhibited Autophagy	(133)
	Cardiomyocytes	lncRNA AK139128 ↑	Cardiac fibroblasts	Promoted apoptosis and inhibited proliferation, migration, and invasion	(134)
	Cardiomyocyte	TGF-beta ↑	RAW264.7 cells	Induced RAW264.7 cells into classically activated macrophages (M1) and M2 macrophages respectively	(135)
	AC16 cardiomyocytes	lncRNA HCG15	Cardiomyocytes	Induced cardiomyocyte apoptosis and the production of inflammatory cytokines	(136)
	Mesenchymal stem cells (MSCs)	HIFs is postulated	Human umbilical vein endothelial cells	Dose-dependent enhancement of *in vitro* proliferation, migration, and tube formation of endothelial cells	(137)
	Adipocyte	Enzymes related to *de novo* lipogenesis ↑, total amount of proteins increased by 3-4-fold ↑	3T3-L1 adipocytes, neighboring preadipocytes and adipocytes	Promoted lipid accumulation, affected lipogenic activity	(138)
	HIF-α-overexpressing donor MSCs (HIF-MSC)	Jagged1 ↑	Endothelial cells	Promoted angiogenic	(139)
	Bone marrow mesenchymal stem cells (BMSCs)	High mobility group box 1 protein (HMGB1) ↑	Human umbilical vein endothelial cells (HUVECs)	Enhancing angiogenesis	(140)
	Bone marrow-derived mesenchymal stem cells (MSCs)	Not mentioned	Infarcted hearts	Improved cardiac function, reduced infarct size and enhanced angiogenesis	(141)
	Human adipose-derived MSCs (hAD-MSCs)	VEGF↑	Human umbilical vein endothelial cells (HUVECs)	Improved angiogenesis	(142)
	Human cardiosphere-derived cells (CDCs)	miR-126 ↑, miR-130a ↑, miR-210 ↑	Human umbilical vein endothelial cells (HUVECs)	Enhancing angiogenesis	(143)
	Cardiac progenitor cells (CPCs)	miR-292↑, miR-210↑, miR-103↑, miR-17↑, miR-20a↑, miR-15b↑, miR-199a↑	Endothelial cells	Enhanced tube formation of endothelial cells and decreased profibrotic gene expression in TGF-β-stimulated fibroblasts	(144)
	Bovine aortic endothelial cells	(IGFBP3, EDN1, CA9, MMP9, VEGFA) ↑, (NAMPT, PLAU, ODC1, EGR1) ↓	Bovine aortic endothelial cells	Increased tube formation	(145)
	Cardiac endothelial cells	HIF ↑, miR-126 ↑, miR-210 ↑	Cardiac progenitor cells (CPCs)	Increased survival of transplanted CPCs in the ischemic heart	(146)
	Cardiac fibroblasts (CFs)	1616 proteins	Cardiomyocyte	Increased myocyte viability under hypoxia	(147)
	Adipose mesenchymal stem cells	Not mentioned	Human umbilical vein endothelial cells (HUVECs)	Promoted neovascularization and graft survival	(148)
	Bone marrow mesenchymal stem cells (BMSCs)	miR-98-5p ↑	Rats with myocardial ischemia-reperfusion injury (MI/RI)	Promoted cardiac function and suppressed myocardial enzyme levels, oxidative stress, inflammation response, macrophage infiltration and infarct size	(149)
	Bone marrow mesenchymal stem cells (BMSCs)	microRNA-24 ↑	H9c2 cells, rats with acute myocardial infarction	decreased the apoptosis rate of H9c2 cells, reduced infarct size and improved cardiac function in acute myocardial infarction rats.	(150)
	Mesenchymal stem cells (MSCs)	miR-210 ↑	Cardiomyocytes, rats with myocardial infarction	Enhanced cardiomyocyte survival, reduced infarct size and improved heart function	(151)
	Human adipose-derived mesenchymal stem cells	lncRNA-MALAT1 ↑	Cardiomyocytes	Protected cardiomyocytes from Dox-induced cardiac damage	(152)
	Adipose mesenchymal stem cells	VEGF ↑, EGF ↑, FGF ↑, VEGF-R2 ↑, VEGF-R3 ↑, MCP-2 ↑, MCP-4 ↑	Human umbilical vein endothelial cells (HUVECs), a nude mouse model of subcutaneous fat grafting	promoted proliferation, migration and tube-formation capability of HUVECs, improved neovascularization around the graft in the nude mouse	(153)
	Bone marrow mesenchymal stem cells (BM-MSCs)	miR-125b-5p ↑	Mice with permanent condition of myocardial infarction (MI)	Exerted a marked cardioprotective function post-MI	(154)
	M1-type macrophage	miR-222	Bone marrow mesenchymal stem cells (BMSCs)	Promoted BMSCs apoptosis	(155)
	Human cardiosphere-derived cells (CDCs)	Not mentioned	Human embryonic stem cell-derived cardiomyocytes	Increase the survival of cardiomyocytes by inhibiting apoptosis	(156)
	C2C12 cells (myotubes)	miR-21 ↑	Mouse tubular epithelial cells (mTECs)	Protected renal tubules from sepsis-induced kidney injury	(157)
Neuron system	Astrocytes	Prion protein ↑	Neurons	Improved survival of neurons	(158)
	Bone marrow mesenchymal stromal cells	NLRP3 ↓, ASC ↓, Caspase-1 ↓, GSDMD-N ↓, cleaved IL-1β↓, IL-18 ↓	Mouse neuroblastoma N2a cells, rat primary cortical neurons	Exhibited significant neuroprotective effects against NLRP3 inflammasome-mediated pyroptosis	(159)
	Mesenchymal stromal cells (MSCs)	miR-21 ↑	APP/PS1 mice	Improved the learning and memory capabilities of APP/PS1 mice,	(160)
	SH-SY5YAPP (695) cells	Aβ40 ↑, Aβ42 ↑	Transgenic APP/PS1 mice	Enhancing the interaction between CD147 and Hook1	(161)
	Microglia	miR-424-5p ↑	Brain microvascular endothelial cells (BMEC)	Aggravated oxygen glucose deprivation (OGD) induced BMEC viability and integrity damage as well as the loss of vascular formation	(162)
	Adipose-Derived Mesenchymal Stem Cells	miR-499a-5p	Neuronal cells, Rats with Spinal Cord Injury	Reduced neuronal apoptosis, reduced cavities formation in the injured area and improved the functional recovery of the hindlimbs of rats	(163)
	Neural progenitor cells	miR-210 ↑	Neural progenitor cells	Increased or inhibited cell viability according to the amount of miR-210	(164)
	SH-SY5Y and HEK293 cells	amyloid-beta (Abeta) ↑	None	Aggravated Alzheimer’s disease (AD)	(165)
	Astrocytes	miR-92b-3p ↑	Neurons	Attenuated oxygen and glucose deprivation-induced neuron death and apoptosis	(166)
Respiratory system	Mesenchymal stromal cell (MSC)	Not mentioned	Mice which exposed to hypoxia after injection	Exerted a pleiotropic protective effect on the lung and inhibit PH through suppression of hyperproliferative pathways, including STAT-3 mediated signaling induced by hypoxia	(167)
	Primary PASMC	miR-143 ↑	Pulmonary artery endothelial cells	Enhanced angiogenesis and cell migration	(168)
	Pulmonary artery endothelial cells (PAEC)	Not mentioned	Pulmonary artery smooth muscle cells (PASMC)	Promoted proliferation and induced apoptosis resistance in PASMC, contributing to the pathogenesis of pulmonary hypertension	(169)
Hematology system	TF-1 cells	miR-486 ↑	TF-1 cells, cord blood CD34+ cells	Induced the erythroid differentiation of TF-1 cells and CD34+ cells	(170)
	K562 cells	A subset of miRNAs (including miR-210) ↑	HUVECs	Significantly enhanced tube formation by HUVECs compared with exosomes produced in normoxic conditions	(171)
	Mesenchymal stem cells	Jagged-1 (Notch ligand) ↑,	Umbilical cord blood hematopoietic stem cells	Enhanced proliferation, increased the self-renewal capacity, quiescence, and clonogenic potential of recipient cells	(172)
Endocrine system	Plasma	Not mentioned	Human naive adipocytes	Promoted the presence of insulin resistance	(3)
	Adipose stem cells	miR-21-3p ↑, miR-126-5p ↑, miR-31-5p ↑, miR-99b ↓, miR-146-a ↓	Diabetic mice	Promoted diabetic wound healing and inhibit inflammation through PI3K/AKT signaling pathway	(173)
	Pericytes	circEhmt1 ↑	Endotheliocytes	Protected endotheliocytes from high glucose induced injury	(174)
	Umbilical cord-derived mesenchymal stem cells	miR-125b ↑	Endothelial cells	Increases endothelial cell proliferation, migration, and inhibited apoptosis, accelerated wound healing	(175)
Urinary system	Injured epithelial cells	TGF-β1 mRNA containing	Fibroblast	Promoted proliferation, alpha-smooth muscle actin expression, F-actin expression, and type I collagen production in fibroblasts.	(4)
	Renal tubular epithelial cells	miR-20a-5p ↑	Renal tubular epithelial cells, mouse model of ischemia-reperfusion-induced acute kidney injury (IRI-AKI)	Inhibition of TECs mitochondrial injury and apoptosis, protected against acute tubular injury	(176)
	Primary renal tubular epithelial cells	miR-21 ↑	Bone marrow-derived dendritic cells (BMDCs)	Promoted the maturation of dendritic cells	(177)
	Renal tubular epithelium cells (NRK-52E)	miR-150-5p ↑	Kidney fibroblasts (NRK-49F), rats with unilateral ischemia reperfusion injury	Activated kidney fibroblasts (NRK-49F), aggravated renal fibrosis	(178)
	Endothelial cell	Lysyl oxidase family member lysyl oxidase-like 2 (LOXL2) ↑	Extracellular matrix (ECM)	Mediated extracellular matrix crosslink and remodelling	(179)
Reproductive system	Placental mesenchymal stem cells (pMSC)	390 proteins	Placental microvascular endothelial cells (hPMEC)	Increased hPMEC migration by 1.6 folds, increased hPMEC tube formation by 7.2 folds; contributed to placental vascular adaptation to low oxygen tension	(180)
	Bovine endometrial stromal cells	128 proteins	Not mentioned	Played a crucial role in maternal-fetal crosstalk and could also affect placental development	(181)
	Cytotrophoblast (CT)	Oxygen-dependent changes of protein	HTR-8/SVneo (EVT)	Promoted EVT invasion and proliferation	(5)
Skeletomuscular system	Mesenchymal stem cell	miR-216a-5p ↑	BV2 microglia *in vitro*; mice with spinal cord injury *in vivo*	Promoted functional behavioral recovery by shifting microglial polarization from M1 to M2 phenotype	(182)
	Mesenchymal stem cells	miR-126 ↑	HUVEC	Promoted angiogenesis, proliferation and migration	(183)
	tenocytes and adjacent adipose-derived mesenchymal stem cells (ADMSCs)	Tenocytes derived exosomes:THSB1, NSEP1, ITIH4 and TN-C. ADMSCs derived exosomes: MMP2, COL6A, CTSD and TN-C	Not mentioned	Involved in multiple signaling pathways of ECM repair and regeneration	(184)
	Polymorphonuclearmyeloid-derived suppressor cells	miR-29a-3p ↑, miR-93-5p ↑	CD4(+) T cell, collagen-induced arthritis (CIA) mouse	Inhibited the proliferation of CD4(+) T, alleviated the arthropathy of CIA mice more effectively	(185)
	Mesenchymal Stem Cells	Not mentioned	Human umbilical vein endothelial cells (HUVECs), rat with steroid-induced osteonecrosis of the femoral head	Promoted angiogenesis and prevented bone loss	(186)
	Synovial fibroblasts (SFs)	miR-424 ↑	T cells	Significantly induced T cells differentiation, which Th17 cells increased and Treg cells decreased	(187)
	Pericytes	Not mentioned	Endothelial cells	Faster wound healing, greater endothelial cord formation in cell culture assays, and greater vascular density in spinal cord tissue	(188)
	Adipose tissue-derived mesenchymal stem/stromal cells (ADSCs)	lncGm37494 ↑	Microglia BV2 cells, mice with spinal cord injury (SCI)	Shifted microglia from M1 to M2 polarization, repaired spinal cord injury	(189)

## Hypoxic Exosomes Promote the Development of Different Types of Tumors

**Figure 2:**
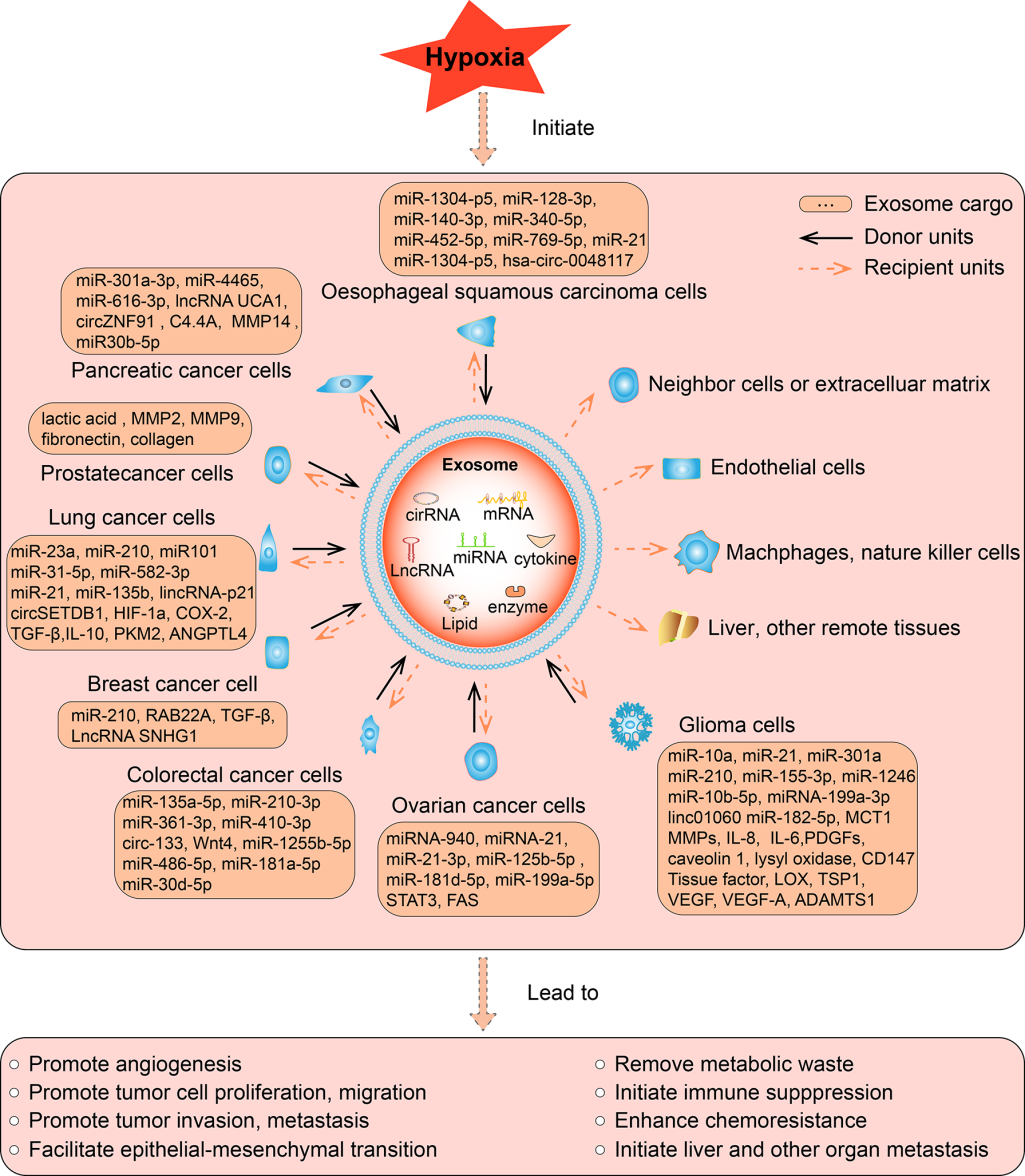
The function of hypoxic exosomes in tumors. In different tumors, hypoxia increased the secretion of exosomes from cancer cells, accompanied by altered expression of its contents, which can be miRNA, lncRNA, cirRNA, mRNA, protein, or lipid. The contents can be upregulated or downregulated in hypoxic exosomes than normoxic exosomes, such contents are briefly listed in the square box. As most studies reported, hypoxia induce the upregulation of its contents, such hypoxic exosomes transmit the key messages to recipient units, result in various biological effects, eventually promote the development of tumors. The solid arrows indicate the donor cells that secret the exosomes, the dashed arrows indicate the recipient cells, organs, and tissues that receive the exosomes. Exosomes are secreted by various hypoxic tumor cells in different types of tumors, and they can be uptaken by normoxic tumor cells, neighboring cells or extracellular matrix, endothelial cells, remote organs or tissues in an autocrine, paracrine, or endocrine manner to exert its function.

Hypoxia is a common phenomenon in the solid tumor microenvironment. The release of exosomes and the contents of exosomes change dramatically under a short supply of oxygen, resulting in altered biological effects. Here, we make a summary of hypoxia induced changes in the cargos and subsequent biological effects of tumor cell derived exosomes in various types of tumor, including glioma, ovarian cancer, breast cancer, prostate cancer, lung cancer, pancreatic cancer, colorectal cancer, liver cancer, oesophageal squamous cell carcinoma, and other types of cancer we retrieved (**Figure 2**).

In glioma, hypoxia promoted the secretion of glioma-derived exosomes and hypoxic exosomes which contain higher expression of miR-10a and miR-21 have a stronger ability than normoxic exosomes to induce myeloid-derived suppressor cells (MDSCs) expansion and potentiate their immunosuppressive functions (39). Hypoxic exosomes isolated from glioblastoma multiforme (GBM) cells have different miRNA and protein quantitative profiles and they were potent inducers of proliferation and angiogenesis *in vivo* and *in vitro*. Moreover, exosomes derived from the hypoxia condition show increased autocrine, promigratory activation of GBM cells (1, 38, 40). Several miRNAs were reported to be upregulated in hypoxic exosomes compared to normoxic exosomes and resulted in further deterioration of glioma. MiR-1246 and miR-10b-5p were enriched in exosomes that produced in the hypoxic microenvironment, and such exosomes were transferred to normoxic glioma cells, promoting their migration and invasion *in vitro* and *in vivo (* 44). Plus, miR-1246 containing exosomes markedly induced M2 macrophage polarization, facilitating the formation of the immunosuppressive microenvironment, thus contributing to promoted glioma proliferation, migration, and invasion *in vitro* and *in vivo (* 48). MiR-155-3p and interleukin 6 (IL-6) were highly expressed in hypoxic glioma-derived exosomes, and such exosomes also induced M2-like macrophage polarization and eventually promotes glioma progression (43). MiR-182-5p expression was significantly upregulated in glioblastoma secreted exosomes in hypoxic conditions. Exosomal miR-182-5p was uptaken by human umbilical vein endothelial cells, leading to KLF2 and KLF4 suppression and VEGFR accumulation, thus promoting tumor angiogenesis (47). miR-301a was also reported to be encapsulated in the hypoxic exosomes dervied from glioblastoma cells, and was transferred to normoxic glioblastoma cells to promote radiation resistance by targeting the tumor suppressor gene TCEAL7 (50). In addition to miRNAs, some proteins were also reported to be increased in the hypoxic exosomes. Hypoxia upregulated the expression of lysyl oxidase homolog 2 and VEGF-F in the glioblastoma derived exosomes, the latter was able to enhance the blood-brain-barrier by interrupting the expression of claudin-5 and occludin (51, 190). Hypoxia also upregulated the monocarboxylate transporter 1(MCT1) and the cluster of differentiation 147 (CD147) in glioma cells and its secreted exosomes, transporting these pro-oncogenic molecules to recipient neighboring cells led to promoted tumor progression (49).

Besides contributing to glioma progression, miRNA containing exosomes also contributed to ischemic injury of peritumoral neurons. MiR-199a-3p was upregulated in a HIF-α dependent way in hypoxic culturing glioma cells derived exosomes. Exosomal miR-199a-3p mediated the oxygen-glucose deprivation (OGD)/reperfusion neuronal injury process by suppressing the mTOR signaling pathway (46). In patients with glioma, serum exosome miR-210 was significantly increased compared to healthy controls. The increased expression level reflected the hypoxic state of glioma patients by positively associated with HIF-α, and it also reflected the changes in malignant glioma loads (42). Apart from miRNA, lncRNA and protein were also encapsulated and transferred by exosomes in hypoxic microenvironment. It was reported that hypoxic glioma stem cells transferred lnc01060 containing exosomes to glioma cells, activating prooncogenic signaling and promoting disease progression. In addition, lnc01060 was upregulated in glioma patients and was significantly correlated with tumor grade and poor clinical prognosis (45). The protein content of secreted exosomes from GBM cells was able to induce differential gene expression in recipient glioma cells. Thus, intercellular communication was facilitated *via* exosomes secreted from hypoxic GBM cells. And prominent changes of genes expression were induced in neighboring normoxic tumor cells and possibly in surrounding stromal cells, many of which are involved in cancer progression and treatment resistance mechanisms (41).

In ovarian cancer, hypoxia also promoted the release of exosomes from cancer cells, and miRNA containing exosomes played a vital role in facilitating tumor progression. MiRNA-940 was upregulated in hypoxic exosomes derived from epithelial ovarian cancer (EOC) (54). EOC cell-derived exosomes delivered miRNAs to induce M2 macrophage polarization, which promoted EOC cell proliferation and migration (56). A similar situation occured in endometrial cancer, the expression of miRNA-21 was increased in exosomes derived from endometrial cancer KLE cells cultured in hypoxia. Exosomal transfer of miRNA-21 to monocyte THP-1 cell promoted its transformation to M2-like polarization macrophages, forming the immune microenvironment in cancer progression (55). In some cases, miRNA was downregulated to facilitate tumor progression. MiR‐199a‐5p was reported to be downregulated in cancer tissue. Hypoxic culturing further decreased the miR‐199a‐5p level in both ovarian cancer cells and their secreted exosomes. The downregulation of miR‐199a‐5p promoted tumor metastasis through targeting HIF-2α to regulate Wnt/β-catenin path-way (53). Besides miRNAs, protein containing exosomes were also involved in ovarian cancer development. It was found that exosomes from hypoxic culturing ovarian cancer cell lines carried more potent oncogenic proteins-STAT3 and FAS, and significantly increased cell migration, invasion, and chemo-resistance *in vitro* and promoted tumor progression, metastasis *in vivo*. In addition, cisplatin efflux *via* exosomes was significantly increased in ovarian cancer cells under hypoxic conditions, contributing to chemo-resistance (52).

In breast cancer, activation of hypoxic signaling also resulted in a significant increase in exosome release. Exposing three different breast cancer cell lines to moderate (1% O_2_) and severe (0.1% O_2_) hypoxia led to a significant increase in the number of exosomes and an increase of miR-210 in exosomes (58). MiR-210 was involved in the expression of vascular remodeling related genes, such as Ephrin A3 and PTP1B, which function to promote angiogenesis (59). In addition to miR-210, lncRNA SNHG1, which was enriched in the hypoxic breast cancer cells secreted exosomes, also promoted the angiogenesis, as well as the proliferation and migration of HUVECs (60). And some research indicated that hypoxic exosomes were preferentially taken up by hypoxic cancer cells (191). The autophagy-associated GPR64 was reported to be upregulated in the hypoxic exosomes derived from the breast cancer associated fibroblast, it stimulated the NF-KB pathway to enhance the invasiveness in recipient breast cancer cells (61). microRNA let-7f was reported to be upregulated both in bone marrow-derived human mesenchymal stem cells (hMSCs) and its secreted exosomes under hypoxic culture, and miR-let-7f attenuated the proliferation and invasion in recipient 4T1 cells (62). In addition to autocrine, hypoxic exosomes also function in a paracrine manner in tumor development. It is reported that under hypoxia, breast cancer cells encapsulate TGF-β into exosomes, such exosomes were able to be taken up by T cells and mediated the suppression of T cell proliferation. Such immunosuppression microenvironment contributes to tumor progression (57). In contrast to cancer cell derived exosomes, natural killer (NK) cells, which play an important role in the tumor immune microenvironment, can also produce exosomes in the tumor microenvironment. Hypoxia enhanced the release of exosomes and increased the expression of three functional proteins of NK cells-specifically FasL, perforin, and granzyme B in hypoxic exosomes. And such hypoxic exosomes exhibited significantly increased cytotoxicity, enhanced inhibition of cell proliferation on breast and pancreatic cancer cells than normoxic exosomes (63).

In prostate cancer, cancer cells secreted more exosomes in a hypoxic microenvironment as a survival mechanism to remove metabolic waste like lactic acid (67). Hypoxia changed not only the secretion amount but also the average size of exosomes secreted by prostate cancer cells. Hypoxic exosomes had a smaller average size and a higher level of exosome biomarkers compared to normoxic exosomes. Co-culturing of hypoxic exosomes with normoxic prostate cancer cells increased the stemness, motility, and invasiveness, promoted prostasphere formation, and enhanced α-SMA expression. Compared to normoxia, hypoxic exosomes showed higher metalloproteinases activity and a higher number of proteins, primarily associated with the remodeling of the epithelial adherens junction pathway (66, 70). Hypoxic exosomes were also involved in cancer metastasis progress. Exosomes derived from hypoxic culturing prostate cancer cells enhanced the level of MMP2, MMP9, and extracellular matrix proteins (fibronectin and collagen) at selective pre-metastatic niches sites, contributing to cancer metastasis (64). Hypoxic exosomes also prompted the development of bladder tumors and renal cell carcinoma (RCC). In bladder tumors, hypoxic exosomes derived from tumor cells showed higher expression levels of lncRNA-UCA1 which could promote tumor growth and progression through epithelial-mesenchymal transition, *in vitro* and *in vivo* (65). In RCC, hypoxia and treatment with CoCl2, a hypoxia mimic agent, increased the CA9 level in exosomes in all RCC cell lines. CA9 exosomes released from hypoxic RCC were postulated to enhance angiogenesis in the microenvironment, thereby contributing to cancer progression (68). MiR-155-5p was found to be upregulated in RCC specimens and hypoxia promoted its selective enrichment in exosomes secreted by hypoxic tumor-associated macrophages (TAM). The exosomes transferred miR-155-5p to RCC and promoted the tumor progression partially through activating IGF1R/PI3K/AKT cascades (71). lncHILAR was also reported to be secreted by hypoxic cancer cells and transferred to normoxic cancer cells through exosomes to activate the miR-613/206/1-1-3p/Jagged-1/Notch/CXCR4 axis, thereby promoting cell invasion and metastasis (69).

In lung cancer, cancer cells produced more exosomes under hypoxic conditions than normoxic conditions. MiRNA, cirRNA, lncRNA, and protein were both reported to be encapsulated in cancer cell derived exosomes and contributed to tumor progression. MiR-23a was significantly upregulated in hypoxic exosomes, resulting in enhanced angiogenesis through regulation of endothelial cells (73). Besides immunosuppressive function, TGF-β, as well as IL-10 which were increased in hypoxic lung cancer cell derived exosomes, also played a positive role in regulating cancer cell migration (74). MiR-31-5p and miR-582-3p were largely internalized within hypoxic exosomes. Exosomal transfer of miRNAs from hypoxic cancer cells to normoxic cancer cells significantly enhanced the proliferation, migration, and invasion of receptive normoxic lung cancer cells *in vitro*, and promoted lung adenocarcinoma metastasis (77, 83). Similarly, hypoxia induced the upregulation of angiopoietin-like 4 (ANGPTL4), HIF-1α/COX-2 and miR-135b and miR-210 in the exosome cargo of A549 cells, exosome transfer of these factors to other A549 cells led to the enhanced proliferation, migration, angiogenesis, and tumor progression (80, 84). In hypoxic tumor microenvironment, bone marrow-derived mesenchymal stem cells (BMSCs) secreted exosomes that contained miR-193a-3p, miR-210-3p and miR-5100, these exosomes were taken up by neighboring epithelial lung cancer cells, led to STAT3 signaling activation and increased expression of mesenchymal related molecules (75). In addition to metastasis and tumor progression, chemoresistance is also involved. Hypoxia upregulated the miR-21 expression in non-small cell lung cancer (NSCLC) cells and cell-derived exosomes, while exosomal miR-21 contributed to cisplatin resistance by downregulating phosphatase and tensin homolog (PTEN) in its recipient normoxic NSCLC cells (81). In some cases, hypoxia decreased the expression of some key genes. It was reported that hypoxic stress suppressed the expression of miR101 in A549 cell secreted exosomes, the exosomes with suppressed expression of miR101 were transferred to THP-1 cells and upregulating the expression of IL-1 and IL-6, contributing to the promoted inflammation of macrophages in the tumor microenvironment (79). Besides miRNA and protein, cirRNA and lncRNA also play a role in tumor progression. circSETDB1 was found to be significantly upregulated in hypoxia-induced exosomes from lung adenocarcinoma (LUAD) cell lines in comparison to normoxic exosomes. And such exosomes improved the migration, invasion, and proliferation capacity of normoxic LUAD cells (78). LncRNA-p21 was reported to be upregulated by hypoxia in non-small cell lung cancer tumor tissue and a higher level of lncRNA-p21 encapsulated exosomes in blood indicates a shorter time to relapse and shorter overall survival. In addition, lncRNA-p21 enriched exosomes promoted tube formation of endothelial cells and enhanced tumor cell adhesion to endothelial cells (76). Besides continuous hypoxia, intermittent hypoxia also has a stronger prosurvival effect than normoxia. Circulating exosomes released under intermittent hypoxia conditions significantly promoted lung carcinoma cells’ malignant properties (72). Hypoxic exosomes also play a role in the chemoresistance of lung cancer therapy. In NSCLC, normoxic and hypoxic exosomes derived from cisplatin-resistant cancer cells were analyzed. As a result, the expression of PKM2 was elevated in hypoxic exosomes. PKM2 promoted glycolysis and finally may neutralize reactive oxygen species (ROS) induced by cisplatin, eventually promoting cisplatin resistance in sensitive NSCLC (82).

In the digestive system malignant disease, including pancreatic cancer, colorectal cancer (CRC), hepatocellular cancer, oesophageal squamous cell carcinoma (OSCC), and other relatively less frequently diagnosed malignant disease like oral squamous cell carcinoma, nasopharyngeal carcinoma, and gastric cancer, hypoxia also exerted a great impact on exosomes and thus contributed to tumor development.

In pancreatic cancer, hypoxic pancreatic cancer cells secreted exosomes that contained miRNA, lncRNA, cirRNA or protein to promote tumor progression. Hypoxia was reported to upregulate a series of miRAN including hsa-miR-29b-3p and hsa-miR-216a-5p,hsa-miR-148a-3p and islet cell damage marker hsa-miR-375 in the exosomes derived from human islets (192). MiR-301a-3p-rich exosomes were induced in the hypoxic microenvironment from pancreatic cancer cells, such exosomes then polarized macrophages to promote malignant behaviors of pancreatic cancer cells (85). miR-30b-5p enriched hypoxic exosomes that derived from pancreatic ductal adenocarcinoma (PDAC) cells, was transferred to HUVEC, leading to enhanced tube formation and angiogenesis (90). LncRNA UCA1 was also reported to be highly expressed in exosomes derived from hypoxic pancreatic cancer cells. Exosomal transfer of lncRNA UCA1 promoted cell migration and tube formation of human umbilical vein endothelial cells (HUVECs), contributing to angiogenesis and tumor growth (89). Interestingly, lncRNA UCA1 was also enriched in exosomes derived from pancreatic stellate cells (PSCs), and such exosome was able to promote the Gemcitabine resistance of pancreatic cancer cells through SOCS3/EZH2 Axis (91). CircZNF91 was encapsulated and transmitted to normoxic pancreatic cancer cells through hypoxic exosomes, eventually enhancing the stability of HIF-1α and leading to glycolysis and chemoresistance of normoxic pancreatic cancer cells (87). C4.4A, a molecular which was upregulated in several tumor types, was upregulated in the pancreatic cancer cells and its secreted exosomes, promoting wound healing and tumor metastasis (86). Pancreatic stellate cells (PSCs), the important components of the tumor microenvironment in pancreatic cancer (PC), also contributed to its development and metastasis through exosome delivery. In tumor hypoxic microenvironment, miR-4465 and miR-616-3p were encapsulated in PSCs secreted exosomes and transmitted to pancreatic cancer cells, promoting PC cell proliferation, migration, and invasion, contributing to PC progression and metastasis (88).

In colorectal cancer (CRC), the hypoxic microenvironment boosted exosome release. Hypoxia promoted colon cancer cells to release more exosomes and thereby promoting self-proliferation in a time-and dose-dependent manner through shortening mitosis duration and upregulating phosphorylated STAT3 (100). Many proteins, miRNAs, cirRNAs were indicated to be upregulated in hypoxic conditions and internalized into exosomes and transferred from hypoxic CRC cancer cells to normoxic cancer cells to promote tumor deterioration. Wnt4, S100A9, MiR-210-3p, miR-361-3p, miR-410-3p, circ-133 were reported to be remarkably elevated in hypoxic CRC exosomes, and can be transferred to normoxic CRC cells, leading to facilitated cell growth and suppressed cell apoptosis, eliciting a protumoral effect, promoting cancer metastasis and enhancing tumor progression. In addition, some miRNA or cirRNA were also enriched in the plasma exosomes of CRC patients and positively associated with a poor prognosis of colorectal cancer (94–98, 103). Some hypoxic colorectal cancer derived exosomes, like exosomes enriched with miR-135a-5p, were phagocytosed by Kupffer cells and transferred from blood circulation into the liver, initiating the large tumor suppressor kinase 2-yes-associated protein-matrix metalloproteinase 7 axis to promote liver metastasis (93). It is worth noting that CRC cells not only secreted exosomes to promote tumor development, but also uptaken exosomes in some case. For example, hypoxic exosomes that was enrich with circEIF3K and derived from cancer-associated fibroblasts (CAF), could be uptaken by colorectal cancer cells (CRC) HCT116 and SW620, and contributed the proliferation, invasion, and tube formation. Knocking down circEIF3K in CAF could mitigate hypoxia induced CRC progression (102). Hypoxic exosomes derived from colorectal cancer cells also enhanced angiogenesis in tumor progression by promoting the proliferation and migration of endothelial cells. Suppression of exosome secretion inhibited these effects (92). In some cases, hypoxia suppressed specific gene expression to adapt to oxygen stress. It was reported that hypoxia downregulated the exosomal miR-1255b-5p that was secreted by colorectal cancer cells and eventually enhanced the epithelial-to-mesenchymal transition as a response to hypoxia (99). Hypoxia also downregulated miR-486-5p and miR-181a-5p while upregulated miR-30d-5p in the cancer cell derived exosomes, both of which were associated with organ-invasiveness and lymph node metastases (101).

In liver cancer, hypoxia promoted tumor self-growth through exosome transfer. Hypoxic exosomes derived from hypoxic hepatocellular carcinoma cancer cells promoted cell proliferation, migration and invasion of normoxic cancer cells through a paracrine manner (111). miR-1273f was reported to be upregulated by hypoxia in the exosomes derived from hepatocellular carcinoma cells, and it was transferred to normoxic cancer cells to promote their proliferation, migration, and invasiveness by targeting LHX6 and subsequently activating Wnt/beta-catenin signaling pathway (108). LncRNA HMMR-AS1 was also increased in the hypoxic exosomes from hepatocellular carcinoma cells, and could be transferred to promote the M2 macrophages polarization and promote the progression of HCC (112). In addition to hepatocellular carcinoma cells, hypoxic tumor exosomes can also be uptaken by HUVECs. It was reported that hypoxia upregulated the miR-155 and miR23a in the hepatocellular carcinoma secreted exosomes. Such exosomes remarkably enhanced tube formation of HUVECs, indicating its facilitating role in promoting angiogenesis in hepatocellular carcinoma (109, 110).

Hypoxia induced significant upregulation of a series of miRNAs in exosomes isolated from oesophageal squamous cell carcinoma (OSCC) cell lines, among which miR-340-5p was reported to be transferred to normoxic OSCC cells through hypoxic exosomes. Such hypoxic exosomes alleviated radiation-induced apoptosis and accelerated DNA damage repair, leading to radioresistance (104). Besides miRNA, hsa-circ-0048117 rich exosomes were also generated in the hypoxic microenvironment in OSCC. Exosomal hsa-circ-0048117 could be transmitted to macrophages to promote M2 polarization and M2 macrophages could enhance the ability of invasion and migration of tumor cells (105). It was reported that hypoxic exosomes derived from esophageal squamous cell carcinoma cells had stronger effects than normoxic exosomes both *in vitro* and *in vivo*. Hypoxic exosomes which contains altered gene information had better effects in promoting proliferation, migration, invasion and tube formation of HUVECs than normoxic exosomes. And hypoxic exosomes also significantly promoted the tumor growth and lung metastasis in nude mice (115). Hypoxic tumor derived exosomes were also reported to influence tumor immune microenvironment. It was reported that the OSCC cell lines: Cal-27 and SCC-9 secreted hypoxic exosomes which contain miR-21 and such exosomes were transferred to myeloid-derived suppressor cells (MDSCs), promoting its suprressive effect on gammadelta T cells, eventually affecting the anti-and pro-tumoral equilibrium (114).

In oral squamous cell carcinoma cells, a hypoxic microenvironment promoted the enrichment of miR-21 in its secreted exosomes. These exosomes were delivered to normoxic cells and promoted metastatic behaviors (113). In nasopharyngeal carcinoma, hypoxic adipocyte-derived exosomes transferred low expression of miR-433-3p into cancer cells, promoting proliferation, migration, and lipid accumulation in cancer cells (107). In many cases, HIF was involved in the pathological process of tumor malignant behaviors. Hypoxic cancer cell derived exosomes can enhance the metastases in a HIF-1α dependent manner. HIF-1α stimulated MMPs expression to affect cell migration and invasion (106). In gastric cancer, hypoxia promoted miR-301a-3p expression in a HIF-α dependent manner and exosomes released from cancer cells were also increased in the hypoxic tumor microenvironment. The miR-301a-3p enriched exosomes were transmitted among gastric cancer cells, eventually leading to cancer cell proliferation, invasion, migration, and epithelial-mesenchymal transition (116).

In many cell types, hypoxia induced the change of RNA and proteins components in cells and such changes were also reflected in hypoxic exosomes (122). In papillary thyroid cancer, hypoxia promoted both the expression of miR-181a in cancer cells and the secretion of miR-181a enriched exosomes from cancer cells. Human umbilical vein endothelial cells (HUVECs) which uptake such exosomes exhibited enhanced proliferation and capillary-like network formation, contributing to tumor angiogenesis (117). In skin carcinoma A431 cells, hypoxia induced the expression of proteins involved in angiogenesis, focal adhesion, extracellular matrix-receptor interaction, and immune cell recruitment. These proteins were encapsulated into exosomes and facilitated angiogenesis and metastasis in the microenvironment (118). In melanoma cells, hypoxia induced the upregulation of 15 miRNAs and downregulation of 3 miRNAs in exosomes derived from cancer cells through miRNA profile analysis (119). Co-culturing of hypoxic exosomes derived from melanoma cells which contained alternated miRNA profiles with THP1 macrophages led to increased M1 markers (CXCL10 and IL6) in macrophages (120). Hypoxia also upregulated the immunomodulatory proteins and chemokines including CSF-1, CCL2, FTH, FTL, and TGFbeta in exosomes from mouse melanoma B16-F0 cells, and such exosomes promoted M2-like polarization of macrophages. And the upregulated miRNA let-7a in hypoxic exosomes enhanced oxidative phosphorylation in macrophages (121). In Ewing’s sarcoma (EWS), hypoxic cancer cells secreted miR-210 enriched exosomes to normoxic cancer cells, leading to promoted sphere formation by targeting the proapoptotic protein CASP8AP2 (123). In multiple myeloma, hypoxia upregulated the expression of miR-1305 in cancer cell secreted exosomes, thus the cellular miR-1305 decreased and its target genes increased, eventually promoting the oncogenic activity of multiple myeloma cells. On the other hand, macrophages uptaken the miR-1305 containing exosomes and exhibited tumor-promoting, M2-macrophage phenotypes (124). miR-135b was also reported to be increased by hypoxia in the exosomes derived from multiple myeloma cells. Such exosomes enhanced endothelial tube formation under hypoxia (125). To conclude, hypoxia increase the release of exosomes from malignant cells and the content of exosomes changed under low oxygen tension, which induce recipient cells to alter gene expression and eventually promote tumor progression.

## Hypoxic Exosomes in Cardiology

### Cardioprotective Effect of Hypoxic Exosomes

Under hypoxia, a series of miRNA were encapsulated into the exosomes and transferred to recipient cells to exhibit cardioprotective effects. A series of cardioprotective miRNAs, including miR-21-5p, miR-378-3p, miR-152-3p, and let-7i-5p were identified in hypoxic exosomes derived from cardiomyocyte H9c2 cells. These anti-apoptotic miRNAs mitigate hypoxia-induced H9c2 cells apoptosis (126). In contrast to these miRNAs, lncRNA HCG15 in the exosomes derived from hypoxic cardiomyocytes led to increased apoptosis, reduced proliferation, and release of inflammatory factors (136). Hypoxia also promoted the accumulation of TGF-beta in exosomes from cardiomyocyte H9c2 cells, and such exosomes could be taken up by RAW264.7 cells and induce the polarization of macrophages and reduce the apoptosis of cardiomyocytes (135). There were large amounts of studies that investigated exosomes derived from mesenchymal stem cells, such exosomes exhibited protective effects in cardiac injury and many miRNAs were reported to be encapsulated in exosome cargo. Especially, exosomes derived from hypoxia pretreated bone marrow-derived mesenchymal stem cells (BMSCs) were able to improve the cardiac function, reduce the infarct size and enhance the angiogenesis of the infarcted hearts (141). miR-98-5p was upregulated by hypoxia in exosomes derived from BMSCs and injection of such exosomes to rats with myocardial ischemia-reperfusion injury improved the cardiac function by targeting TRL4 and activating PI3K/AKT signaling pathway (149). miR-24 in exosomes derived from BMSCs was also upregulated by hypoxia, and such exosomes decreased the apoptosis rate of H9c2 cells, reduced infarct sized and improved cardiac function in acute myocardial infarction rats (150). miR-210 in exosomes derived from mesenchymal stem cells was increased under hypoxic culture, administration of such exosomes enhanced cardiomyocyte survival to hypoxia *in vitro*, reduced infarct size and improved heart function *in vivo (* 151). Injecting such hypoxic exosomes into the infarcted heart of C57BL/6 mouse resulted in significantly higher survival, smaller scar size, and better cardiac functions recovery. Hypoxic exosomes conferred increased vascular density, lower cardiomyocytes apoptosis, reduced fibrosis, and increased recruitment of cardiac progenitor cells in the infarcted heart relative to exosomes isolated from the same cell line cultured under normoxia (127). miR-125b-5p in the exosomes from mesenchymal stem cells was also upregulated by hypoxia, adminstration of such exosomes to mice with myocardial infarction suppressed the expression of the proapoptotic genes p53 and BAK1 in cardiomyocytes and exerted a marked cardioprotective function post-infarction (154). Hypoxic exosomes derived from human cardiosphere-derived cells also exhibited protective effects in cardiomyocytes by inhibiting apoptosis (156).

Although most miRNAs were upregulated in hypoxic exosomes, some miRNAs were enriched in normoxic exosomes rather than hypoxic exosomes. miR-10b-5p was reported to be downregulated in hypoxic exosomes derived from endothelial colony forming cells compared to normoxic exosomes. Due to a reduction of miR-10b-5p, which targets the fibrotic genes Smurf1 and HDAC4, the anti-fibrotic effects of exosomes were abolished (128). Besides continuous hypoxia, intermittent hypoxia also alters endothelial cell derived exosome cargo, including has-mir-383-3p, and such exosomes promote increased permeability and dysfunction of endothelial cells, contributing to cardiovascular dysfunction (129). Apart from miRNAs, lncRNA and cirRNA are also important non-coding RNAs that are induced and transferred through hypoxic exosomes. LncRNA-UCA1 is elevated both in myocardial infarction patients and exosomes derived from hypoxic culturing mesenchymal stem cells (hMSCs). Intramyocardial injection of lncRNA-UCA1 containing exosomes to rats with myocardial infarction demonstrated that hypoxic exosomes had a better cardioprotection effect than normoxic exosomes (130). lncRNA-MALAT1 was increased by hypoxia in exosomes from human adipose-derived mesenchymal stem cells, such exosomes functioned as competing endogenous RNAs (ceRNAs) binding to miR-92a-3p to protect cardiomyocyte from doxorubicin (Dox) induced cardiac damage (152). CirRNA is also involved in cardioprotective effects. circHIPK3 expression was found to be significantly upregulated in hypoxic exosomes compared to normoxic exosomes. Such circHIPK3 encapsulated exosomes were transferred from cardiomyocytes to cardiac microvascular endothelial cells (CMVECs). Leading to promoted endothelial cell migration, proliferation, and tube formation *in vitro*, and can effectively reduce the infarct area and promote angiogenesis in the border surrounding the infarcted area in myocardial infarction mice (132). Hypoxia also upregulated a series of proteins, like vascular endothelial growth factor (VEGF), epidermal growth factor (EGF), fibroblast growth factor (FGF) and their receptors (VEGF-R2, VEGF-R3), and monocyte chemoattractant protein 2 (MCP-2), monocyte chemoattractant protein 4 (MCP-4) in the exosomes derived from adipose mesenchymal stem cells, administration of such hypoxic exosomes promoted proliferation, migration, and tube-formation capability of HUVECs, and also improved neovascularization around the graft in the nude mice model of fat grafting (153).

In addition to protective effects, the hypoxia augmented miRNA in exosomes can also be harmful in some cases. MiR-30a was highly enriched in exosomes either from the culture medium of cardiomyocytes after hypoxic stimulation *in vitro* or the serum of acute myocardial infarction (AMI) patients *in vivo*. The miR-30a enriched exosomes were regulated by HIF-α and were efficiently transferred between cardiomyocytes in an autocrine manner after hypoxia. But miR-30a inhibits the expression of core autophagy regulators, which are beneficial in ischemic heart disease. Inhibition of such exosomes release or inhibition of miR-30a was considered a promising treatment strategy in AMI (133). miR-222 was also reported to be transferred under hypoxic culture from M1-type macrophages to bone marrow mesenchymal stem cells, and co-culturing with such exosomes led to decreased cell viability, migration and increased apoptosis in the recipient cells, such effects were partly abolished by the exosome secretion inhibitor GM4869. Similarly, lncRNA AK139128 was also increased in hypoxia and played a negative role. Cardiomyocyte derived exosomal which contained lncRNA AK139128 promoted apoptosis and inhibited proliferation, migration, and invasion in cardiac fibroblasts. Such exosomes could also exacerbate myocardial infarction in the rat model (134).

### Hypoxic Exosomes Promote Angiogenesis in Cardiology

Upon hypoxia, exosomes and microvesicles released by bone marrow mesenchymal stem cells (MSCs) were easily taken up by human umbilical vein endothelial cells (HUVECs), which led to promoted angiogenesis and improved cardiac function in infarction attack (137). The total amount of proteins secreted from exosomes increased by 3-4 folds under hypoxic conditions (138). Overexpressing HIF-1α in MSCs led to an increased angiogenic capacity mediated by its secreted exosomes (139). High mobility group box 1 protein (HMGB1) was induced by hypoxia in MSCs derived exosomes. Exosomal HMGB1 transferred to HUVECs activated JNK signaling and induced HIF-1α dependent VEGF expression, eventually led to enhanced angiogenesis (140). Compared with normoxia, hypoxic exosomes were more easily to be taken up by HUVECs and their angiogenesis stimulatory activity was also significantly enhanced. The expression of vascular endothelial growth factor (VEGF) and activation of the protein kinase A (PKA) signaling pathway in HUVECs was significantly increased by hypoxia-exposed exosomes (142). Besides, HUVECs also uptaken exosomes derived from hypoxic culturing human cardiosphere derived cells and exhibited enhanced tube formation. Pro-angiogenic exosomal miRNAs including miR-126, miR-130a, and miR-210 showed a substantial increase in the hypoxic exosomes compared to normoxic exosomes (143).

Besides bone marrow meschmenal stem cells, cardiac progenitor cells (CPCs), and other stem cell types were also attractive candidates for the treatment of myocardial infarction, however, the role of exosomes in the treatment remains unclear. Recent research shows that upon hypoxia, exosomes secreted by cardiac progenitor cells enhanced tube formation of endothelial cells and decreased profibrotic gene expression in TGF-β-stimulated fibroblasts. Microarray analysis identified 11 miRNAs that were upregulated in hypoxic exosomes compared with normoxic exosomes. Hypoxic exosomes improved cardiac function and reduced fibrosis (144). In research involving different degrees of hypoxia, human CPCs were cultured under normoxia (21% O_2_), physoxia (5% O_2_), and hypoxia (1% O_2_) conditions. As a result, the release of exosomes under physoxia increased 1.6 folds and significantly increased tube formation compared to normoxia and hypoxia (145). Interestingly, exosomes secreted by endothelial cells cultured under hypoxia can be taken up by CPCs, giving them increased tolerance when subjected to *in vitro* hypoxic stress. These exosomes overexpress HIF1 and have higher contents of miR-126 and miR-210 which activated prosurvival kinases and induced a glycolytic switch in recipient cells (146). These studies revealed close crosstalk between cardiac progenitor cells and endothelial cells *via* exosomes. For stem cell derived exosomes, the stemness of the cell is crucial for the treatment effect. In research evaluating the effect of donor’s age and hypoxia to exosomes, cardiac progenitor cells secreted exosomes from older children were reparative only when subjected to hypoxic conditions (193).

## Hypoxic Exosomes in Non-Malignant Disease From Different Systems

**Figure 3:**
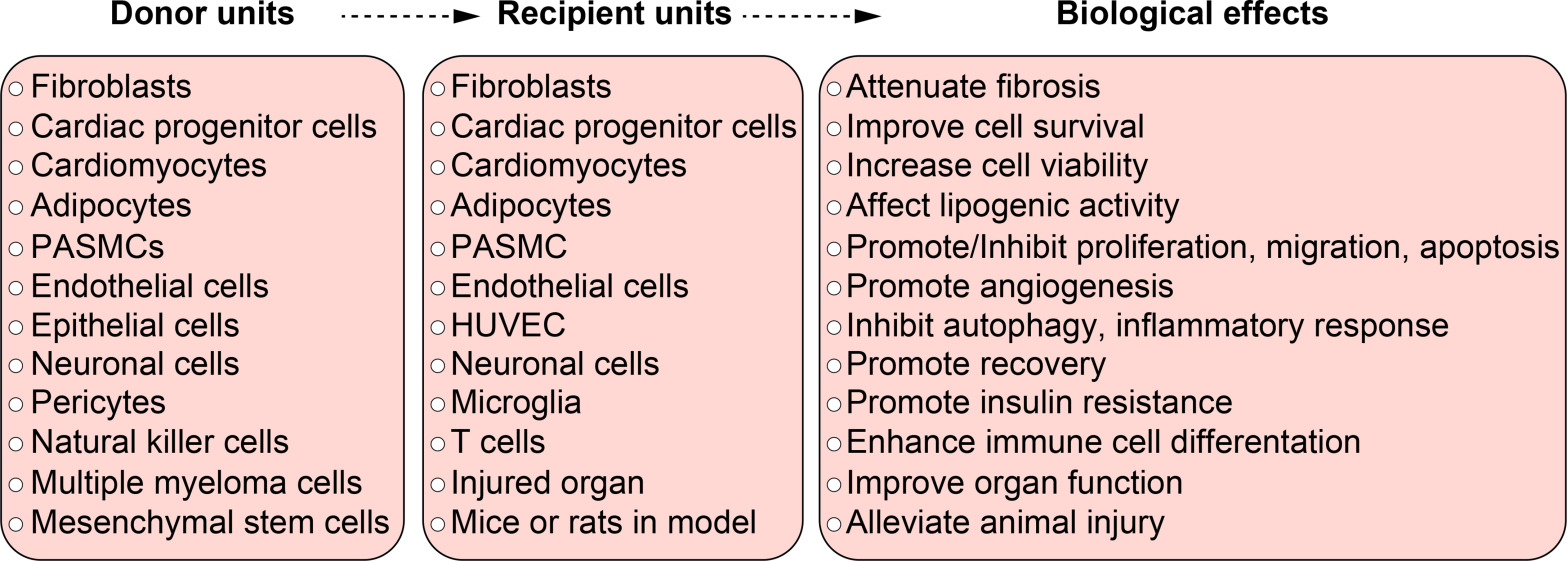
The function of hypoxic exosomes in non-malignant diseases. A summary of donor units, recipient units, and biological effects in different systems. Each box briefly summarizes the corresponding contents from different systems, they don’t relate to each other line to line. Specific relationship among the donor units, recipient units, and biological effects can be found in **Table 1** and in the body text.

Besides cardiovascular system, hypoxic exosomes also play a comprehensive role in diseases from other systems. A wide range of donor units and recipient units are involved, and various biological effects are induced (**Figure 3**).

### Neuron System

In the neuron system, different hypoxic exosomal content has been reported and they play a neuron protective role from different perspectives. Amyloid-β (Aβ), which is related to the pathogenesis of Alzheimer’s disease, was accumulated during a hypoxic environment. Hypoxia increased the level of Aβ40 and Aβ42 in human neuroblastoma SH-SY5Y cells derived exosomes and such exosomes promote amyloids propagation (161). Hypoxia also increased Amyloid-beta (Aβ) both in the extracellular and exosomes of HEK293, which aggravated Alzheimer’s disease (AD) (165). Prion protein in astrocytes is a sensor for oxidative stress and mediates beneficial cellular responses. Under hypoxic and ischemic conditions, the release of exosomes carrying enriched prion protein and other molecules led to improved survival of neurons (158). The expression of NLRP3, ASC, Caspase-1, GSDMD-N, cleaved IL-1β and IL-18 were decreased in neuron cells after taking up bone marrow mesenchymal stromal cells-derived exosomes. Hypoxic exosomes had more significant effects to decrease these molecules, and exhibited a more pronounced neuroprotective effect against oxygen-glucose deprivation injury (159). MiR-21 was also reported to be increased by hypoxia in exosomes derived from mesenchymal stromal cells and mediated the improvement of neurologic conditions. And hypoxic exosomes were demonstrated to have better effects than normoxic exosomes to ameliorate cognitive decline in APP/PS1 mice by rescuing synaptic dysfunction and regulating inflammatory responses (160). miR-92b-3p was reported to be upregulated in exosomes derived from oxygen-glucose deprivated (OGD) astrocytes, such exosomes could be uptaken by neurons and attenuated OGD-induced neuron death and apoptosis (166). miR-499a-5p that was encapsulated into exosomes from hypoxic adipose tissue-derived stromal cells (ADSCs) reduced neuronal apoptosis after oxygen-glucose deprivation and reperfusion (OGD/R) *in vitro*, and relieved the damage of rats with spinal cord injury (SCI) *in vivo (* 163).

Apart from the protective role, hypoxic exosomal content can be harmful as well. MiR-424-5p was upregulated in the exosomes derived from oxygen-glucose deprivation activated microglia. Such exosomes induced significant cell damage and permeability of microvascular endothelial cells. This interpreted how microglia secreted exosomes participate in neurovascular unit injury under the ischemic and hypoxic state (162). miR-210 was upregulated by hypoxia in exosomes from neural stem/progenitor cells, but high levels of miR-210 inhibited the cell viability of recipient neural stem/progenitor cells (164).

### Respiratory System

Hypoxia is a broad accepted factor to induce vascular remodeling and the development of pulmonary hypertension. Hypoxic exosomes from different donor cells play a different role in this process. Hypoxia and inflammation could induce pulmonary artery endothelial cells (PAECs) to release exosomes. These exosomes were involved in overproliferation and apoptosis resistance in pulmonary arterial smooth muscle cells (PASMCs), by which they may contribute to the pathogenesis of pulmonary hypertension (169). In contrast, hypoxia can also induce PASMCs to release exosomes and the exosomes were transferred to PAECs in a paracrine manner, affecting the migration and apoptosis of PAECs, then promoting the development of pulmonary arterial hypertension (168). Exosomes transferred between PAECs and PASMCs promoted the development of pulmonary hypertension while exosomes derived from mesenchymal stromal cells prevented the development of pulmonary hypertension. Hypoxic mesenchymal stromal cells derived exosomes exerted a pleiotropic protective effect on the lung and inhibit hypoxic pulmonary hypertension through suppression of hyperproliferative pathways, including STAT-3 mediated signaling induced by hypoxia (2).

### Hematology System

Hypoxia enhanced the package of miRNA and protein in exosomes derived from hematology system and such exosomes could be taken up by endothelial cells and leading to enhanced tube formation, or they could be taken up by other hematology cells and promoting further differentiation. Compared to normoxia, hypoxia significantly upregulated a subset of miRNAs, including miR-210, in exosomes derived from human leukemia cells. Coculturing such exosomes with HUVECs remarkably enhanced tube formation by HUVECs compared with exosomes produced in normoxic conditions (171). MiR-486 was also upregulated by hypoxia both in intracellular space and in the secreted exosomes of erythroleukemia TF-1 cells. Exosomal miR-486 played critical roles in regulating the hypoxia-induced erythroid differentiation of hematopoietic cells (170). Hypoxia also upregulated the the Jagged-1 (Notch ligand) in the exosomes from mesenchymal stem cells, and co-culture such exosomes with hematopoietic stem cells (HSCs) enhanced the proliferation, increased the self-renewal capacity, quiescence, and clonogenic potential of HSCs (172).

### Endocrine System

Hypoxic exosomes play a bilateral role in diabetic disease. On one hand, hypoxic exosomes led to insulin resistance in some cases. Exosomes isolated from sleep fragmentation and intermittent hypoxia exposed mice induced attenuated p-AKT/total AKT responses to exogenous insulin, leading to insulin resistance in adipocytes (3). On the other hand, hypoxic exosomes internalized miRNA or lncRNA to exert a protective role in diabetic disease. Compared to normoxia, miR-21-3p, miR-126-5p, miR-31-5p were significantly upregulated while miR-99b and miR-146-a were significantly downregulated in hypoxic exosomes derived from adipose stem cells. Hypoxic exosomes exhibited significantly better effects in promoting wound healing than normoxic exosomes when they were injected into diabetic mice (173). miR-125b was also upregulated by hypoxia in the exosomes from umbilical cord-derived mesenchymal stem cells (ucMSCs), such exosomes can be taken up by endothelial cells and promoted cell proliferation, migration, inhibited apoptosis, and accelerated wound healing by targeting and suppressing the tumor protein p53 inducible nuclear protein 1 (TP53INP1) (175). In addition, it was reported that hypoxia upregulated circEhmt1 both in pericytes and its secreted exosomes. CircEhmt1 that were encapsulated into exosomes were transmitted to endotheliocytes, protecting endotheliocytes against high glucose induced injury (174). In another word, hypoxic exosomes mediated impaired insulin sensitivity in the intermittent hypoxia process.

### Urinary System

In the urinary system, hypoxia did not change the average size of exosomes secreted by rat renal proximal tubular cells, but significantly increased exosome production in a time-dependent manner. HIF-1 induction also promoted exosome secretion, pharmacological and genetic suppression of HIF-1 abrogated the increase of exosome secretion under hypoxia (194). Many miRNAs were reported to be encapsulated in the exosomes from tubular epithelial cells and exhibited renal protective effects both *in vivo* and *in vitro*. Hypoxia induced the enrichment of miR-20a-5p in exosomes from renal tubular epithelial cells. Such exosomes were protective in acute tubular injury by promoting tubular epithelial cells proliferation and improving mitochondrial functions (176). Hypoxia increased the miR-21 both in tubular epithelial cells and in the exosomes of bone marrow-derived dendritic cells. And it was postulated that the upregulated miR-21 of tubular epithelial cells promoted dentritic cell maturation (177). Hypoxia upregulated the miR-150-5p in the exosomes from rat renal tubular epithelium cells (NRK-52E), such exosomes could activate rat kidney fibroblasts (NRK-49F), and injection of such exosomes alleviated renal fibrosis in rats with unilateral ischemia reperfusion injury (178). Hypoxia also upregulated the miR-21 in the exosomes from myotubes C2C12 cells, and such exosomes protected renal tubes from sepsis-induced kidney injury (157). In addition to tubular epithelial cells, exosomes from epithelial cells were also more active under hypoxia. Hypoxia promoted epithelial cells to produce an increased number of exosomes containing TGF-β1 mRNA, to activate fibroblasts, contributing to fibrosis that involved in the pathology of hypoxia induced kidney injury (4). Hypoxia upregulated the lysyl oxidase family member lysyl oxidase-like 2 (LOXL2) in the exosomes from endothelial cells, and such exosomes mediated extracellular matrix crosslink and remodelling (179).

### Reproductive System

Different degree of hypoxia has different impacts on exosome contents. Compared to the controls (8% O_2_), exosomes released from placental mesenchymal stem cells increased by 3.3 folds in 1% O_2_ and 6.7 folds in 3% O_2_ respectively. Such changes may contribute to placental vascular adaptation to low oxygen tension under both physiological and pathological conditions (180). Similarly, in endometrial stromal cells, the secretion of exosomes cultured at 1% O_2_ was increased by about 3.6 folds compared with 8% O_2_. Mass spectrometry analysis identified 128 proteins unique to exosomes produced at 1% O_2_ compared with only 46 proteins unique to those produced at 8% O_2_. Differential production of proteins was associated with different specific biological processes (181). It was reported that hypoxic cytotrophoblast derived exosomes promoted extravillous trophoblasts’ invasion and proliferation (5). Implying the mechanism of hypoxic placenta contributing to the pathophysiology of preeclampsia. Besides proteins encapsulated in hypoxic exosomes, hypoxic exosomal miRNA may also contribute to the pathogenesis of preeclampsia. The level of circulating exosomal total-miRNA and the hypoxia sensitive hsa-miR-210 was elevated in preeclampsia and it was higher in the severe form. Hsa-miR-210 was secreted *via* exosomes, which may play a role in the pathomechanism of the disease (195).

### Skeletomuscular System

In the skeletomuscular system, hypoxic exosomes play an important role to promote angiogenesis and injury recovery in different joint damage. In the spinal cord injury model, hypoxia resulted in an enrichment of miR-216a-5p in the exosomes from mesenchymal stem cells (MSCs). Hypoxic exosomes promoted functional behavioral recovery by shifting microglial polarization from M1 to M2 phenotype *in vivo* and *in vitro (* 182). LncGm37494 was also reported to be upregulated by hypoxia in the exosomes from adipose tissue-derived mesenchymal stem/stromal cells, and such hypoxic exosomes were more effective than normoxic exosomes in repairing spinal cord injury (189). In spinal cord angiogenesis, preconditioning pericyte with hypoxia mimic and coculture it with endothelial cells resulted in faster wound healing, greater endothelial cord formation, and greater vascular density in the spinal cord tissue. Exosome secretion and the physical presence of stimulated pericytes were necessary for the promotion of angiogenic outcomes (188). In a research of bone fracture healing, hypoxia enhanced the production of exosomal miR-126 through the activation of HIF-1α. Such exosomes were transferred to HUVECs and promoted angiogenesis, proliferation, and migration *in vitro*. *In vivo* experiments demonstrated that hypoxic exosome administration promoted bone fracture healing through exosomal miR-126 transfer (183). In rotator cuff tendon injuries (RCTI), the hypoxic environment triggered tenocytes and adjacent adipose-derived mesenchymal stem cells (ADMSCs) to release exosomes to the extracellular matrix (ECM). Tenocytes secreted exosomes encapsulated THSB1, NSEP1, ITIH4, and TN-C. ADMSCs secreted exosomes encapsulated MMP2, COL6A, CTSD, and TN-C. These proteins were involved in multiple signaling pathways of ECM repair and regeneration, protecting the tendon matrix from injury (184). In the arthropathy of collagen-induced arthritis (CIA), hypoxia upregulated the expression of miR-29a-3p and miR-93-5p in exosomes derived from polymorphonuclear myeloid-derived suppressor cells. Compared to normoxic exosomes, hypoxic exosomes could inhibit the proliferation of CD4(+) T cells more effectively. And the administration of hypoxic exosomes alleviated mice with CIA more effectively (185). In steroid-induced osteonecrosis of the femoral head (ONFH) model, hypoxic exosomes derived from bone marrow mesenchymal stem cells (BMMSCs) exhibited better therapeutic effects in promoting angiogenesis and preventing bone loss (186). In rheumatoid arthritis, hypoxia induced the upregulation of miR-424 in exosomes derived from synovial fibroblasts. Exosomal miR-424 could significantly induce T cells differentiation and contribute to the aggravation of rheumatoid arthritis (187).

## Conclusion

In many hypoxia related diseases, miRNAs have been reported most to be internalized into exosomes under hypoxia. The master regulator of oxygen homeostasis and a hypoxia sensitive transcription factor HIF, has been reported to have crosstalk with miRNA by many studies. HIF-1α/HIF-1β dimmer can bind to the promoters of target miRNAs and regulate extensive cellular processes, including proliferation, metastasis, apoptosis, and drug resistance, etc. Also, tumor-related miRNAs can modulate the activity of HIF-1α, and play a positive or negative role in tumor development (196). Whether HIF regulated miRNAs are more easily to be encapsulated into exosomes and if there is crosstalk between HIF and other lncRNAs, cirRNAs, proteins, and Lipids that internalized into hypoxic exosomes still awaits further investigation.

As mentioned above, in various types of tumors and other diseases, hypoxia not only increases the quantities of exosome release but also alters the contents of exosomes. Since exosomes are a crucial mediator of genetic, protein, and lipid information carrier and transmitter, manipulating the release, degradation, cargo sorting, and receiving of exosomes becomes a promising strategy to interfere with disease development. In tumor development, hypoxic tumor cells secret more exosomes, such hypoxic exosomes disseminate the malignant information to recipient cells, tissue, and organs, contributing to tumor progression. Reducing exosomes release, promoting lysosome degradation, inhibiting key factors encapsulating, or disrupting exosomes uptaking could be a possible way to prevent the invasion and metastasis of tumors. In fact, through high-throughput screening, a series of inhibitors and activators of exosomes biogenesis have been identified in prostate cancer cells (197). And the mechanisms of how inhibitors function have been well reviewed (198, 199). But targeting exosome donor cells exploration, a broader range of cell line study and the *in vivo* effects investigation is warranted. Vice versa, when exosomes are beneficial, for example, exosomes derived from mesenchymal stem cells play a cardioprotective role and promote angiogenesis in acute myocardial infarction, and prevent the development of pulmonary hypertension. Under such circumstances, facilitating the secretion and function of exosomes is profitable for improving health conditions. In fact, there are already attempt to applying exosomes in cancer therapy. Reprocessing exosomes that target immune cells to induce an immune response against the tumor, as well as other infectious diseases has been investigated by researchers (200). Such strategies were also explored in several tumor types, including colorectal cancer, metastatic melanoma, and non-small cell lung cancer (201). More research is needed to extend such kind of engineer for disease control.

In addition, although the release of exosomes is increased under hypoxia, there is also baseline release of exosomes under a physiological state. In another word, almost all cell types within the body produce exosomes and the mixed exosomes exist in almost all intercellular space and body fluids. The increased amount of exosome secretion and some specific encapsulated markers make exosomes a potential biomarker for detecting tumors. The closed and naturally biocompatible membrane structure, stable circulation in the blood, and ability to cross the blood-brain-barrier make exosomes ideal candidates to deliver drugs or genes to target spots (202, 203). But how to classify the exosomes, distinguish their parent cells, identify their target cells remains less elucidated. More research is needed to investigate the specific molecular markers of exosomes to categorize them. Elucidating the specific mechanism of exosomes network and function has great potential for deep understanding and accurate interferer with disease progression.

To conclude, hypoxia increased the release of exosomes in tumor development and other diseases. Donor cells grown in hypoxic microenvironment release exosomes that encapsulate miRNA, lncRNA, circRNA, mRNA, cytokines, and enzymes and transfer them to recipient cells, tissue or, organs in an autocrine, paracrine, or endocrine manner. Such exosomal transfer eventually leads to promoted angiogenesis, tumor progression and metastasis, epithelial meschemenal transition, metabolic waste disposal, immune suppression, and chemoresistance in tumor development. In non-malignant disease, hypoxic exosomes can lead to attenuated fibrosis, altered cell proliferation/migration/apoptosis, autography and inflammation inhibition, immune cell differentiation, insulin resistance, injury recovery, and organ function improvement. Understanding the biogenesis, sorting, and receiving of exosomes provide new perspectives to interfere with the disease.

## Author Contributions

HJ, writing—original draft. HZ and MZ, writing—discussing about the ideas in the draft. YH and XCL, writing—review and editing. YX and XSL, resources, funding acquisition, project administration, and writing—review and editing. All authors contributed to the article and approved the submitted version.

## Funding

This study was funded by grants from the National Natural Science Foundation of China (No. 81973987, 81700051 and 81700052).

## Conflict of Interest

The authors declare that the research was conducted in the absence of any commercial or financial relationships that could be construed as a potential conflict of interest.

## Publisher’s Note

All claims expressed in this article are solely those of the authors and do not necessarily represent those of their affiliated organizations, or those of the publisher, the editors and the reviewers. Any product that may be evaluated in this article, or claim that may be made by its manufacturer, is not guaranteed or endorsed by the publisher.

## Glossary

**Table:**

HIFs	Hypoxia Induced Factors
PHD	prolyl hydroxylases
HREs	hypoxia-responsive elements
ESCRT	endosomal sorting complex required for transport
MDSCs	myeloid-derived suppressor cells
GBM	glioblastoma multiforme
IL-6	interleukin 6
RORA	RAR-related orphan receptor alpha
PTEN	phosphatase and tensin homolog
MCT1	monocarboxylate transporter 1
CD147	cluster of differentiation 147
OGD	oxygen-glucose deprivation
EOC	epithelial ovarian cancer
hMSCs	human mesenchymal stem cells
NK	natural killer
RCC	renal cell carcinoma
TAM	tumor-associated macrophages
ANGPTL4	angiopoietin-like 4
BMSCs	bone marrow-derived mesenchymal stem cells
PTEN	phosphatase and tensin homolog
LUAD	lung adenocarcinoma
NSCLC	non-small cell lung cancer
ROS	reactive oxygen species
CRC	colorectal cancer
OSCC	oesophageal squamous cell carcinoma
HUVECs	human umbilical vein endothelial cells
PSCs	Pancreatic stellate cells
PC	pancreatic cancer
OSCC	oesophageal squamous cell carcinoma
MDSCs	myeloid-derived suppressor cells
HUVECs	Human umbilical vein endothelial cells
EWS	Ewing’s sarcoma
BMSCs	bone marrow mesenchymal stem cells
ceRNAs	competing endogenous RNAs
Dox	doxorubicin
CMVECs	cardiac microvascular endothelial cells
VEGF	vascular endothelial growth factor
EGF	epidermal growth factor
FGF	fibroblast growth factor
MCP	monocyte chemoattractant protein
AMI	acute myocardial infarction
MSCs	mesenchymal stem cells
HUVECs	human umbilical vein endothelial cells
HMGB1	High mobility group box 1 protein
PKA	protein kinase A
CPCs	cardiac progenitor cells
Aβ	Amyloid-β
AD	Alzheimer’s disease
PAECs	pulmonary artery endothelial cells
PASMCs	pulmonary arterial smooth muscle cells
HSCs	hematopoietic stem cells
ucMSCs	umbilical cord-derived mesenchymal stem cells
TP53INP1	tumor protein p53 inducible nuclear protein 1
LOXL2	lysyl oxidase-like 2
MSCs	mesenchymal stem cells
RCTI	rotator cuff tendon injuries
ADMSCs	adjacent adipose-derived mesenchymal stem cells
ECM	extracellular matrix
CIA	collagen-induced arthritis
ONFH	osteonecrosis of the femoral head
BMMSCs	bone marrow mesenchymal stem cells
PDAC	pancreatic ductal adenocarcinoma
PSCs	pancreatic stellate cells
ADSCs	adipose tissue-derived stromal cells
OGD/R	oxygen-glucose deprivation and reperfusion
SCI	spinal cord injury

## References

[1] Kucharzewska P Christianson HC Welch JE Svensson KJ Fredlund E Ringner M. Exosomes Reflect the Hypoxic Status of Glioma Cells and Mediate Hypoxia-Dependent Activation of Vascular Cells During Tumor Development. Proc Natl Acad Sci USA (2013) 110:7312–7. doi: 10.1073/pnas.1220998110 PMC3645587 23589885

[2] Lee C Mitsialis SA Aslam M Vitali SH Vergadi E Konstantinou G. Exosomes Mediate the Cytoprotective Action of Mesenchymal Stromal Cells on Hypoxia-Induced Pulmonary Hypertension. Circulation (2012) 126:2601–11. doi: 10.1161/CIRCULATIONAHA.112.114173 PMC3979353 23114789

[3] Khalyfa A Gozal D Masa JF Marin JM Qiao Z Corral J. Sleep-Disordered Breathing, Circulating Exosomes, and Insulin Sensitivity in Adipocytes. Int J Obes (Lond) (2018) 42:1127–39. doi: 10.1038/s41366-018-0099-9 PMC6195831 29892042

[4] Borges FT Melo SA Ozdemir BC Kato N Revuelta I Miller CA. TGF-Beta1-Containing Exosomes From Injured Epithelial Cells Activate Fibroblasts to Initiate Tissue Regenerative Responses and Fibrosis. J Am Soc Nephrol (2013) 24:385–92. doi: 10.1681/ASN.2012101031 PMC3582210 23274427

[5] Salomon C Kobayashi M Ashman K Sobrevia L Mitchell MD Rice GE. Hypoxia-Induced Changes in the Bioactivity of Cytotrophoblast-Derived Exosomes. PloS One (2013) 8:e79636. doi: 10.1371/journal.pone.0079636 24244532 PMC3823597

[6] Semenza GL. Hypoxia-Inducible Factors in Physiology and Medicine. Cell (2012) 148:399–408. doi: 10.1016/j.cell.2012.01.021 22304911 PMC3437543

[7] Noman MZ Hasmim M Messai Y Terry S Kieda C Janji B. Hypoxia: A Key Player in Antitumor Immune Response. A Review in the Theme: Cellular Responses to Hypoxia. Am J Physiol Cell Physiol (2015) 309:C569–79. doi: 10.1152/ajpcell.00207.2015 PMC4628936 26310815

[8] Majmundar AJ Wong WJ Simon MC. Hypoxia-Inducible Factors and the Response to Hypoxic Stress. Mol Cell (2010) 40:294–309. doi: 10.1016/j.molcel.2010.09.022 20965423 PMC3143508

[9] Schönenberger MJ. Hypoxia Signaling Pathways: Modulators of Oxygen-Related Organelles. Front Cell Dev Biol (2015) 3. doi: 10.3389/fcell.2015.00042 PMC4508581 26258123

[10] Keith B Johnson RS Simon MC. HIF1α and HIF2α: Sibling Rivalry in Hypoxic Tumour Growth and Progression. Nat Rev Cancer (2011) 12:9–22. doi: 10.1038/nrc3183 22169972 PMC3401912

[11] Pugh CW Ratcliffe PJ. New Horizons in Hypoxia Signaling Pathways. Exp Cell Res (2017) 356:116–21. doi: 10.1016/j.yexcr.2017.03.008 PMC5653532 28315322

[12] Choudhry H Harris AL. Advances in Hypoxia-Inducible Factor Biology. Cell Metab (2018) 27:281–98. doi: 10.1016/j.cmet.2017.10.005 29129785

[13] Goodall GJ Wickramasinghe VO. RNA in Cancer. Nat Rev Cancer (2021) 21:22–36. doi: 10.1038/s41568-020-00306-0 33082563

[14] Shao C Yang F Miao S Liu W Wang C Shu Y. Role of Hypoxia-Induced Exosomes in Tumor Biology. Mol Cancer (2018) 17:120. doi: 10.1186/s12943-018-0869-y 30098600 PMC6087002

[15] Shao H Im H Castro CM Breakefield X Weissleder R Lee H. New Technologies for Analysis of Extracellular Vesicles. Chem Rev (2018) 118:1917–50. doi: 10.1021/acs.chemrev.7b00534 PMC6029891 29384376

[16] Couch Y Buzas EI Vizio DD Gho YS Harrison P Hill AF. A Brief History of Nearly EV-Erything - The Rise and Rise of Extracellular Vesicles. J Extracell Vesicles (2021) 10:e12144. doi: 10.1002/jev2.12144 34919343 PMC8681215

[17] Witwer KW Thery C. Extracellular Vesicles or Exosomes? On Primacy, Precision, and Popularity Influencing a Choice of Nomenclature. J Extracell Vesicles (2019) 8:1648167. doi: 10.1080/20013078.2019.1648167 31489144 PMC6711079

[18] Gould SJ Raposo G. As We Wait: Coping With an Imperfect Nomenclature for Extracellular Vesicles. J Extracell Vesicles (2013) 2:10.3402. doi: 10.3402/jev.v2i0.20389 PMC3760635 24009890

[19] Klumperman J Raposo G. The Complex Ultrastructure of the Endolysosomal System. Cold Spring Harb Perspect Biol (2014) 6:a16857. doi: 10.1101/cshperspect.a016857 PMC4176003 24851870

[20] Thebaud B Stewart DJ. Exosomes: Cell Garbage can, Therapeutic Carrier, or Trojan Horse? Circulation (2012) 126:2553–5. doi: 10.1161/CIRCULATIONAHA.112.146738 23114790

[21] Pan BT Teng K Wu C Adam M Johnstone RM. Electron Microscopic Evidence for Externalization of the Transferrin Receptor in Vesicular Form in Sheep Reticulocytes. J Cell Biol (1985) 101:942–8. doi: 10.1083/jcb.101.3.942 PMC2113705 2993317

[22] Harding C Heuser J Stahl P. Receptor-Mediated Endocytosis of Transferrin and Recycling of the Transferrin Receptor in Rat Reticulocytes. J Cell Biol (1983) 97:329–39. doi: 10.1083/jcb.97.2.329 PMC2112509 6309857

[23] Huotari J Helenius A. Endosome Maturation. EMBO J (2011) 30:3481–500. doi: 10.1038/emboj.2011.286 PMC3181477 21878991

[24] Hurley JH Hanson PI. Membrane Budding and Scission by the ESCRT Machinery: It’s All in the Neck. Nat Rev Mol Cell Biol (2010) 11:556–66. doi: 10.1038/nrm2937 PMC2922035 20588296

[25] Luzio JP Gray SR Bright NA. Endosome-Lysosome Fusion. Biochem Soc Trans (2010) 38:1413–6. doi: 10.1042/BST0381413 21118098

[26] Colombo M Raposo G Thery C. Biogenesis, Secretion, and Intercellular Interactions of Exosomes and Other Extracellular Vesicles. Annu Rev Cell Dev Biol (2014) 30:255–89. doi: 10.1146/annurev-cellbio-101512-122326 25288114

[27] Thery C. Exosomes: Secreted Vesicles and Intercellular Communications. F1000 Biol Rep (2011) 3:15. doi: 10.3410/B3-15 21876726 PMC3155154

[28] Pan BT Johnstone RM. Fate of the Transferrin Receptor During Maturation of Sheep Reticulocytes *In Vitro*: Selective Externalization of the Receptor. Cell (1983) 33:967–78. doi: 10.1016/0092-8674(83)90040-5 6307529

[29] Nagarajah S. Exosome Secretion - More Than Simple Waste Disposal? Implications for Physiology, Diagnostics and Therapeutics. J Circ Biomark (2016) 5:7. doi: 10.5772/62975 28936255 PMC5548323

[30] Yanez-Mo M Siljander PR Andreu Z Zavec AB Borras FE Buzas EI. Biological Properties of Extracellular Vesicles and Their Physiological Functions. J Extracell Vesicles (2015) 4:27066. doi: 10.3402/jev.v4.27066 25979354 PMC4433489

[31] Milane L Singh A Mattheolabakis G Suresh M Amiji MM. Exosome Mediated Communication Within the Tumor Microenvironment. J Control Release (2015) 219:278–94. doi: 10.1016/j.jconrel.2015.06.029 26143224

[32] Thery C Boussac M Veron P Ricciardi-Castagnoli P Raposo G Garin J. Proteomic Analysis of Dendritic Cell-Derived Exosomes: A Secreted Subcellular Compartment Distinct From Apoptotic Vesicles. J Immunol (2001) 166:7309–18. doi: 10.4049/jimmunol.166.12.7309 11390481

[33] van Niel G D’Angelo G Raposo G. Shedding Light on the Cell Biology of Extracellular Vesicles. Nat Rev Mol Cell Biol (2018) 19:213–28. doi: 10.1038/nrm.2017.125 29339798

[34] Li Q Huang Q Huyan T Wang Y Huang Q Shi J. Bifacial Effects of Engineering Tumour Cell-Derived Exosomes on Human Natural Killer Cells. Exp Cell Res (2018) 363:141–50. doi: 10.1016/j.yexcr.2017.12.005 29269076

[35] Banizs AB Huang T Nakamoto RK Shi W He J. Endocytosis Pathways of Endothelial Cell Derived Exosomes. Mol Pharm (2018) 15:5585–90. doi: 10.1021/acs.molpharmaceut.8b00765 PMC6465166 30351959

[36] Mulcahy LA Pink RC Carter DR. Routes and Mechanisms of Extracellular Vesicle Uptake. J Extracell Vesicles (2014) 3:10.3402. doi: 10.3402/jev.v3.24641 PMC4122821 25143819

[37] Yaghoubi S Najminejad H Dabaghian M Karimi MH Abdollahpour-Alitappeh M Rad F. How Hypoxia Regulate Exosomes in Ischemic Diseases and Cancer Microenvironment? IUBMB Life (2020) 72:1286–305. doi: 10.1002/iub.2275 32196941

[38] Svensson KJ Kucharzewska P Christianson HC Skold S Lofstedt T Johansson MC. Hypoxia Triggers a Proangiogenic Pathway Involving Cancer Cell Microvesicles and PAR-2-Mediated Heparin-Binding EGF Signaling in Endothelial Cells. Proc Natl Acad Sci USA (2011) 108:13147–52. doi: 10.1073/pnas.1104261108 PMC3156184 21788507

[39] Guo X Qiu W Liu Q Qian M Wang S Zhang Z. Immunosuppressive Effects of Hypoxia-Induced Glioma Exosomes Through Myeloid-Derived Suppressor Cells *via* the miR-10a/Rora and miR-21/Pten Pathways. Oncogene (2018) 37:4239–59. doi: 10.1038/s41388-018-0261-9 29713056

[40] Zhang G Zhang Y Cheng S Wu Z Liu F Zhang J. CD133 Positive U87 Glioblastoma Cells-Derived Exosomal microRNAs in Hypoxia- Versus Normoxia-Microenviroment. J Neurooncol (2017) 135:37–46. doi: 10.1007/s11060-017-2566-x 28948499

[41] Kore RA Edmondson JL Jenkins SV Jamshidi-Parsian A Dings R Reyna NS. Hypoxia-Derived Exosomes Induce Putative Altered Pathways in Biosynthesis and Ion Regulatory Channels in Glioblastoma Cells. Biochem Biophys Rep (2018) 14:104–13. doi: 10.1016/j.bbrep.2018.03.008 PMC5986551 29872742

[42] Lan F Yue X Xia T. Exosomal microRNA-210 is a Potentially Non-Invasive Biomarker for the Diagnosis and Prognosis of Glioma. Oncol Lett (2020) 19:1967–74. doi: 10.3892/ol.2020.11249 PMC7039075 32194691

[43] Xu J Zhang J Zhang Z Gao Z Qi Y Qiu W. Hypoxic Glioma-Derived Exosomes Promote M2-Like Macrophage Polarization by Enhancing Autophagy Induction. Cell Death Dis (2021) 12:373. doi: 10.1038/s41419-021-03664-1 33828078 PMC8026615

[44] Qian M Chen Z Guo X Wang S Zhang Z Qiu W. Exosomes Derived From Hypoxic Glioma Deliver miR-1246 and miR-10b-5p to Normoxic Glioma Cells to Promote Migration and Invasion. Lab Invest (2021) 101:612–24. doi: 10.1038/s41374-020-00522-0 33446893

[45] Li J Liao T Liu H Yuan H Ouyang T Wang J. Hypoxic Glioma Stem Cell-Derived Exosomes Containing Linc01060 Promote Progression of Glioma by Regulating the MZF1/c-Myc/HIF1α Axis. Cancer Res (2021) 81:114–28. doi: 10.1158/0008-5472.CAN-20-2270 33158815

[46] Zhao JL Tan B Chen G Che XM Du ZY Yuan Q. Hypoxia-Induced Glioma-Derived Exosomal miRNA-199a-3p Promotes Ischemic Injury of Peritumoral Neurons by Inhibiting the mTOR Pathway. Oxid Med Cell Longev (2020) 2020:5609637. doi: 10.1155/2020/5609637 33110474 PMC7578720

[47] Li J Yuan H Xu H Zhao H Xiong N. Hypoxic Cancer-Secreted Exosomal miR-182-5p Promotes Glioblastoma Angiogenesis by Targeting Kruppel-Like Factor 2 and 4. Mol Cancer Res (2020) 18:1218–31. doi: 10.1158/1541-7786.MCR-19-0725 32366676

[48] Qian M Wang S Guo X Wang J Zhang Z Qiu W. Hypoxic Glioma-Derived Exosomes Deliver microRNA-1246 to Induce M2 Macrophage Polarization by Targeting TERF2IP *via* the STAT3 and NF-kappaB Pathways. Oncogene (2020) 39:428–42. doi: 10.1038/s41388-019-0996-y 31485019

[49] Thakur A Qiu G Xu C Han X Yang T Ng SP. Label-Free Sensing of Exosomal MCT1 and CD147 for Tracking Metabolic Reprogramming and Malignant Progression in Glioma. Sci Adv (2020) 6:z6119. doi: 10.1126/sciadv.aaz6119 PMC7319757 32637597

[50] Yue X Lan F Xia T. Hypoxic Glioma Cell-Secreted Exosomal miR-301a Activates Wnt/beta-Catenin Signaling and Promotes Radiation Resistance by Targeting Tceal7. Mol Ther (2019) 27:1939–49. doi: 10.1016/j.ymthe.2019.07.011 PMC6838947 31402274

[51] Zhao C Wang H Xiong C Liu Y. Hypoxic Glioblastoma Release Exosomal VEGF-A Induce the Permeability of Blood-Brain Barrier. Biochem Biophys Res Commun (2018) 502:324–31. doi: 10.1016/j.bbrc.2018.05.140 29787762

[52] Dorayappan K Wanner R Wallbillich JJ Saini U Zingarelli R Suarez AA. Hypoxia-Induced Exosomes Contribute to a More Aggressive and Chemoresistant Ovarian Cancer Phenotype: A Novel Mechanism Linking STAT3/Rab Proteins. Oncogene (2018) 37:3806–21. doi: 10.1038/s41388-018-0189-0 PMC6043362 29636548

[53] Lian XY Zhang H Liu Q Lu X Zhou P He SQ. Ovarian Cancer-Excreted Exosomal miR-199a-5p Suppresses Tumor Metastasis by Targeting Hypoxia-Inducible Factor-2alpha in Hypoxia Microenvironment. Cancer Commun (Lond) (2020) 40:380–5. doi: 10.1002/cac2.12034 PMC7427304 32428376

[54] Chen X Ying X Wang X Wu X Zhu Q Wang X. Exosomes Derived From Hypoxic Epithelial Ovarian Cancer Deliver microRNA-940 to Induce Macrophage M2 Polarization. Oncol Rep (2017) 38:522–8. doi: 10.3892/or.2017.5697 28586039

[55] Xiao L He Y Peng F Yang J Yuan C. Endometrial Cancer Cells Promote M2-Like Macrophage Polarization by Delivering Exosomal miRNA-21 Under Hypoxia Condition. J Immunol Res (2020) 2020:9731049. doi: 10.1155/2020/9731049 33110923 PMC7579677

[56] Chen X Zhou J Li X Wang X Lin Y Wang X. Exosomes Derived From Hypoxic Epithelial Ovarian Cancer Cells Deliver microRNAs to Macrophages and Elicit a Tumor-Promoted Phenotype. Cancer Lett (2018) 435:80–91. doi: 10.1016/j.canlet.2018.08.001 30098399

[57] Rong L Li R Li S Luo R. Immunosuppression of Breast Cancer Cells Mediated by Transforming Growth Factor-Beta in Exosomes From Cancer Cells. Oncol Lett (2016) 11:500–4. doi: 10.3892/ol.2015.3841 PMC4727188 26870240

[58] King HW Michael MZ Gleadle JM. Hypoxic Enhancement of Exosome Release by Breast Cancer Cells. BMC Cancer (2012) 12:421. doi: 10.1186/1471-2407-12-421 22998595 PMC3488584

[59] Jung KO Youn H Lee CH Kang KW Chung JK. Visualization of Exosome-Mediated miR-210 Transfer From Hypoxic Tumor Cells. Oncotarget (2017) 8:9899–910. doi: 10.18632/oncotarget.14247 PMC5354779 28038441

[60] Dai G Yang Y Liu S Liu H. Hypoxic Breast Cancer Cell-Derived Exosomal SNHG1 Promotes Breast Cancer Growth and Angiogenesis *via* Regulating miR-216b-5p/JAK2 Axis. Cancer Manag Res (2022) 14:123–33. doi: 10.2147/CMAR.S327621 PMC8751978 35027847

[61] Xi L Peng M Liu S Liu Y Wan X Hou Y. Hypoxia-Stimulated ATM Activation Regulates Autophagy-Associated Exosome Release From Cancer-Associated Fibroblasts to Promote Cancer Cell Invasion. J Extracell Vesicles (2021) 10:e12146. doi: 10.1002/jev2.12146 34545708 PMC8452512

[62] Egea V Kessenbrock K Lawson D Bartelt A Weber C Ries C. Let-7f miRNA Regulates SDF-1alpha- and Hypoxia-Promoted Migration of Mesenchymal Stem Cells and Attenuates Mammary Tumor Growth Upon Exosomal Release. Cell Death Dis (2021) 12:516. doi: 10.1038/s41419-021-03789-3 34016957 PMC8137693

[63] Jiang Y Jiang H Wang K Liu C Man X Fu Q. Hypoxia Enhances the Production and Antitumor Effect of Exosomes Derived From Natural Killer Cells. Ann Transl Med (2021) 9:473. doi: 10.21037/atm-21-347 33850870 PMC8039676

[64] Deep G Jain A Kumar A Agarwal C Kim S Leevy WM. Exosomes Secreted by Prostate Cancer Cells Under Hypoxia Promote Matrix Metalloproteinases Activity at Pre-Metastatic Niches. Mol Carcinog (2020) 59:323–32. doi: 10.1002/mc.23157 PMC7189745 31943365

[65] Xue M Chen W Xiang A Wang R Chen H Pan J. Hypoxic Exosomes Facilitate Bladder Tumor Growth and Development Through Transferring Long Non-Coding RNA-Uca1. Mol Cancer (2017) 16:143. doi: 10.1186/s12943-017-0714-8 28841829 PMC5574139

[66] Ramteke A Ting H Agarwal C Mateen S Somasagara R Hussain A. Exosomes Secreted Under Hypoxia Enhance Invasiveness and Stemness of Prostate Cancer Cells by Targeting Adherens Junction Molecules. Mol Carcinog (2015) 54:554–65. doi: 10.1002/mc.22124 PMC4706761 24347249

[67] Panigrahi GK Praharaj PP Peak TC Long J Singh R Rhim JS. Author Correction: Hypoxia-Induced Exosome Secretion Promotes Survival of African-American and Caucasian Prostate Cancer Cells. Sci Rep (2018) 8:6645. doi: 10.1038/s41598-018-24997-6 29691455 PMC5915453

[68] Horie K Kawakami K Fujita Y Sugaya M Kameyama K Mizutani K. Exosomes Expressing Carbonic Anhydrase 9 Promote Angiogenesis. Biochem Biophys Res Commun (2017) 492:356–61. doi: 10.1016/j.bbrc.2017.08.107 28851650

[69] Hu G Ma J Zhang J Chen Y Liu H Huang Y. Hypoxia-Induced lncHILAR Promotes Renal Cancer Metastasis *via* ceRNA for the miR-613/206/1-1-3p/Jagged-1/Notch/CXCR4 Signaling Pathway. Mol Ther (2021) 29:2979–94. doi: 10.1016/j.ymthe.2021.05.020 PMC8531137 34058384

[70] Panigrahi GK Ramteke A Birks D Abouzeid AH Venkataraman S Agarwal C. Exosomal microRNA Profiling to Identify Hypoxia-Related Biomarkers in Prostate Cancer. Oncotarget (2018) 9:13894–910. doi: 10.18632/oncotarget.24532 PMC5862624 29568403

[71] Gu W Gong L Wu X Yao X. Hypoxic TAM-Derived Exosomal miR-155-5p Promotes RCC Progression Through HuR-Dependent IGF1R/AKT/PI3K Pathway. Cell Death Discov (2021) 7:147. doi: 10.1038/s41420-021-00525-w 34131104 PMC8206073

[72] Almendros I Khalyfa A Trzepizur W Gileles-Hillel A Huang L Akbarpour M. Tumor Cell Malignant Properties Are Enhanced by Circulating Exosomes in Sleep Apnea. Chest (2016) 150:1030–41. doi: 10.1016/j.chest.2016.08.1438 27568581

[73] Hsu YL Hung JY Chang WA Lin YS Pan YC Tsai PH. Hypoxic Lung Cancer-Secreted Exosomal miR-23a Increased Angiogenesis and Vascular Permeability by Targeting Prolyl Hydroxylase and Tight Junction Protein ZO-1. Oncogene (2017) 36:4929–42. doi: 10.1038/onc.2017.105 28436951

[74] Wang Y Yi J Chen X Zhang Y Xu M Yang Z. The Regulation of Cancer Cell Migration by Lung Cancer Cell-Derived Exosomes Through TGF-Beta and IL-10. Oncol Lett (2016) 11:1527–30. doi: 10.3892/ol.2015.4044 PMC4734314 26893774

[75] Zhang X Sai B Wang F Wang L Wang Y Zheng L. Hypoxic BMSC-Derived Exosomal miRNAs Promote Metastasis of Lung Cancer Cells *via* STAT3-Induced EMT. Mol Cancer (2019) 18:40. doi: 10.1186/s12943-019-0959-5 30866952 PMC6417285

[76] Castellano JJ Marrades RM Molins L Vinolas N Moises J Canals J. Extracellular Vesicle lincRNA-P21 Expression in Tumor-Draining Pulmonary Vein Defines Prognosis in NSCLC and Modulates Endothelial Cell Behavior. Cancers (Basel) (2020) 12:734. doi: 10.3390/cancers12030734 PMC7140053 32244977

[77] Yu F Liang M Huang Y Wu W Zheng B Chen C. Hypoxic Tumor-Derived Exosomal miR-31-5p Promotes Lung Adenocarcinoma Metastasis by Negatively Regulating SATB2-Reversed EMT and Activating MEK/ERK Signaling. J Exp Clin Cancer Res (2021) 40:179. doi: 10.1186/s13046-021-01979-7 34074322 PMC8167983

[78] Xu L Liao WL Lu QJ Zhang P Zhu J Jiang GN. Hypoxic Tumor-Derived Exosomal Circular RNA SETDB1 Promotes Invasive Growth and EMT *via* the miR-7/Sp1 Axis in Lung Adenocarcinoma. Mol Ther Nucleic Acids (2021) 23:1078–92. doi: 10.1016/j.omtn.2021.01.019 PMC7875767 33614250

[79] Li J Xu P Wu D Guan M Weng X Lu Y. Hypoxic Stress Suppresses Lung Tumor-Secreted Exosomal Mir101 to Activate Macrophages and Induce Inflammation. Cell Death Dis (2021) 12:776. doi: 10.1038/s41419-021-04030-x 34362882 PMC8346509

[80] Chen J Xu R Xia J Huang J Su B Wang S. Aspirin Inhibits Hypoxia-Mediated Lung Cancer Cell Stemness and Exosome Function. Pathol Res Pract (2019) 215:152379. doi: 10.1016/j.prp.2019.03.008 30878308

[81] Dong C Liu X Wang H Li J Dai L Li J. Hypoxic Non-Small-Cell Lung Cancer Cell-Derived Exosomal miR-21 Promotes Resistance of Normoxic Cell to Cisplatin. Onco Targets Ther (2019) 12:1947–56. doi: 10.2147/OTT.S186922 PMC6420102 30881046

[82] Wang D Zhao C Xu F Zhang A Jin M Zhang K. Cisplatin-Resistant NSCLC Cells Induced by Hypoxia Transmit Resistance to Sensitive Cells Through Exosomal PKM2. Theranostics (2021) 11:2860–75. doi: 10.7150/thno.51797 PMC7806469 33456577

[83] Wang J Zhao J Zhu J Zhang S. Hypoxic Non-Small-Cell Lung Cancer Cell-Secreted Exosomal microRNA-582-3p Drives Cancer Cell Malignant Phenotypes by Targeting Secreted Frizzled-Related Protein 1. Cancer Manag Res (2020) 12:10151–61. doi: 10.2147/CMAR.S263768 PMC7569064 33116870

[84] Mo F Xu Y Zhang J Zhu L Wang C Chu X. Effects of Hypoxia and Radiation-Induced Exosomes on Migration of Lung Cancer Cells and Angiogenesis of Umbilical Vein Endothelial Cells. Radiat Res (2020) 194:71–80. doi: 10.1667/RR15555.1 32352864

[85] Wang X Luo G Zhang K Cao J Huang C Jiang T. Hypoxic Tumor-Derived Exosomal miR-301a Mediates M2 Macrophage Polarization *via* PTEN/PI3Kgamma to Promote Pancreatic Cancer Metastasis. Cancer Res (2018) 78:4586–98. doi: 10.1158/0008-5472.CAN-17-3841 29880482

[86] Ngora H Galli UM Miyazaki K Zoller M. Membrane-Bound and Exosomal Metastasis-Associated C4.4A Promotes Migration by Associating With the Alpha(6)Beta(4) Integrin and MT1-MMP. Neoplasia (2012) 14:95–107. doi: 10.1593/neo.111450 22431918 PMC3306255

[87] Zeng Z Zhao Y Chen Q Zhu S Niu Y Ye Z. Hypoxic Exosomal HIF-1alpha-Stabilizing CircZNF91 Promotes Chemoresistance of Normoxic Pancreatic Cancer Cells via Enhancing Glycolysis. Oncogene (2021) 40:5505–17. doi: 10.1038/s41388-021-01960-w 34294845

[88] Cao W Zeng Z He Z Lei S. Hypoxic Pancreatic Stellate Cell-Derived Exosomal Mirnas Promote Proliferation and Invasion of Pancreatic Cancer Through the PTEN/AKT Pathway. Aging (Albany NY) (2021) 13:7120–32. doi: 10.18632/aging.202569 PMC7993707 33653966

[89] Guo Z Wang X Yang Y Chen W Zhang K Teng B. Hypoxic Tumor-Derived Exosomal Long Noncoding RNA UCA1 Promotes Angiogenesis *via* miR-96-5p/AMOTL2 in Pancreatic Cancer. Mol Ther Nucleic Acids (2020) 22:179–95. doi: 10.1016/j.omtn.2020.08.021 PMC7498711 32942233

[90] Chen K Wang Q Liu X Wang F Yang Y Tian X. Hypoxic Pancreatic Cancer Derived Exosomal miR-30b-5p Promotes Tumor Angiogenesis by Inhibiting GJA1 Expression. Int J Biol Sci (2022) 18:1220–37. doi: 10.7150/ijbs.67675 PMC8771853 35173549

[91] Chi Y Xin H Liu Z. Exosomal lncRNA UCA1 Derived From Pancreatic Stellate Cells Promotes Gemcitabine Resistance in Pancreatic Cancer *via* the SOCS3/EZH2 Axis. Front Oncol (2021) 11:671082. doi: 10.3389/fonc.2021.671082 34868904 PMC8640181

[92] Huang Z Feng Y. Exosomes Derived From Hypoxic Colorectal Cancer Cells Promote Angiogenesis Through Wnt4-Induced Beta-Catenin Signaling in Endothelial Cells. Oncol Res (2017) 25:651–61. doi: 10.3727/096504016X14752792816791 PMC7841118 27712599

[93] Sun H Meng Q Shi C Yang H Li X Wu S. Hypoxia-Inducible Exosomes Facilitate Liver-Tropic Premetastatic Niche in Colorectal Cancer. Hepatology (2021) 74:2633–51. doi: 10.1002/hep.32009 34110633

[94] Ge L Zhou F Nie J Wang X Zhao Q. Hypoxic Colorectal Cancer-Secreted Exosomes Deliver miR-210-3p to Normoxic Tumor Cells to Elicit a Protumoral Effect. Exp Biol Med (Maywood) (2021) 246:1895–906. doi: 10.1177/15353702211011576 PMC8424635 33969722

[95] Li J Yang P Chen F Tan Y Huang C Shen H. Hypoxic Colorectal Cancer-Derived Extracellular Vesicles Deliver microRNA-361-3p to Facilitate Cell Proliferation by Targeting TRAF3 *via* the Noncanonical NF-kappaB Pathways. Clin Transl Med (2021) 11:e349. doi: 10.1002/ctm2.349 33784010 PMC7967919

[96] Yang H Zhang H Yang Y Wang X Deng T Liu R. Hypoxia Induced Exosomal circRNA Promotes Metastasis of Colorectal Cancer *via* Targeting GEF-H1/RhoA Axis. Theranostics (2020) 10:8211–26. doi: 10.7150/thno.44419 PMC7381736 32724467

[97] Hu X Mu Y Liu J Mu X Gao F Chen L. Exosomes Derived From Hypoxic Colorectal Cancer Cells Transfer miR-410-3p to Regulate Tumor Progression. J Cancer (2020) 11:4724–35. doi: 10.7150/jca.33232 PMC7330706 32626519

[98] Huang Z Yang M Li Y Yang F Feng Y. Exosomes Derived From Hypoxic Colorectal Cancer Cells Transfer Wnt4 to Normoxic Cells to Elicit a Prometastatic Phenotype. Int J Biol Sci (2018) 14:2094–102. doi: 10.7150/ijbs.28288 PMC6299371 30585272

[99] Zhang X Bai J Yin H Long L Zheng Z Wang Q. Exosomal miR-1255b-5p Targets Human Telomerase Reverse Transcriptase in Colorectal Cancer Cells to Suppress Epithelial-to-Mesenchymal Transition. Mol Oncol (2020) 14:2589–608. doi: 10.1002/1878-0261.12765 PMC7530775 32679610

[100] Ren R Sun H Ma C Liu J Wang H. Colon Cancer Cells Secrete Exosomes to Promote Self-Proliferation by Shortening Mitosis Duration and Activation of STAT3 in a Hypoxic Environment. Cell Biosci (2019) 9:62. doi: 10.1186/s13578-019-0325-8 31402975 PMC6683569

[101] Bjornetro T Redalen KR Meltzer S Thusyanthan NS Samiappan R Jegerschold C. An Experimental Strategy Unveiling Exosomal microRNAs 486-5p, 181a-5p and 30d-5p From Hypoxic Tumour Cells as Circulating Indicators of High-Risk Rectal Cancer. J Extracell Vesicles (2019) 8:1567219. doi: 10.1080/20013078.2019.1567219 30728923 PMC6352936

[102] Yang K Zhang J Bao C. Exosomal Circeif3k From Cancer-Associated Fibroblast Promotes Colorectal Cancer (CRC) Progression *via* miR-214/PD-L1 axis. BMC Cancer (2021) 21:933. doi: 10.1186/s12885-021-08669-9 34412616 PMC8375187

[103] Wang Y Yin K Tian J Xia X Ma J Tang X. Granulocytic Myeloid-Derived Suppressor Cells Promote the Stemness of Colorectal Cancer Cells Through Exosomal S100a9. Adv Sci (Weinh) (2019) 6:1901278. doi: 10.1002/advs.201901278 31559140 PMC6755519

[104] Chen F Xu B Li J Yang X Gu J Yao X. Hypoxic Tumour Cell-Derived Exosomal miR-340-5p Promotes Radioresistance of Oesophageal Squamous Cell Carcinoma *via* KLF10. J Exp Clin Cancer Res (2021) 40:38. doi: 10.1186/s13046-021-01834-9 33485367 PMC7825246

[105] Lu Q Wang X Zhu J Fei X Chen H Li C. Hypoxic Tumor-Derived Exosomal Circ0048117 Facilitates M2 Macrophage Polarization Acting as miR-140 Sponge in Esophageal Squamous Cell Carcinoma. Onco Targets Ther (2020) 13:11883–97. doi: 10.2147/OTT.S284192 PMC7682796 33239890

[106] Shan Y You B Shi S Shi W Zhang Z Zhang Q. Hypoxia-Induced Matrix Metalloproteinase-13 Expression in Exosomes From Nasopharyngeal Carcinoma Enhances Metastases. Cell Death Dis (2018) 9:382. doi: 10.1038/s41419-018-0425-0 29515112 PMC5841433

[107] Yin H Qiu X Shan Y You B Xie L Zhang P. HIF-1alpha Downregulation of miR-433-3p in Adipocyte-Derived Exosomes Contributes to NPC Progression *via* Targeting SCD1. Cancer Sci (2021) 112:1457–70. doi: 10.1111/cas.14829 PMC8019221 33511729

[108] Yu Y Min Z Zhou Z Linhong M Tao R Yan L. Hypoxia-Induced Exosomes Promote Hepatocellular Carcinoma Proliferation and Metastasis *via* miR-1273f Transfer. Exp Cell Res (2019) 385:111649. doi: 10.1016/j.yexcr.2019.111649 31562861

[109] Matsuura Y Wada H Eguchi H Gotoh K Kobayashi S Kinoshita M. Exosomal miR-155 Derived From Hepatocellular Carcinoma Cells Under Hypoxia Promotes Angiogenesis in Endothelial Cells. Dig Dis Sci (2019) 64:792–802. doi: 10.1007/s10620-018-5380-1 30465177

[110] Sruthi TV Edatt L Raji GR Kunhiraman H Shankar SS Shankar V. Horizontal Transfer of miR-23a From Hypoxic Tumor Cell Colonies can Induce Angiogenesis. J Cell Physiol (2018) 233:3498–514. doi: 10.1002/jcp.26202 28929578

[111] Zou M You Y He S Wu XL. Effects of Hypoxic Exosomes on the Proliferation, Migration and Invasion of Hepatocellular Carcinoma Huh7 Cells. Zhonghua Gan Zang Bing Za Zhi (2019) 27:363–8. doi: 10.3760/cma.j.issn.1007-3418.2019.05.008 PMC12769755 31177661

[112] Wang X Zhou Y Dong K Zhang H Gong J Wang S. Exosomal lncRNA HMMR-AS1 Mediates Macrophage Polarization Through miR-147a/ARID3A Axis Under Hypoxia and Affects the Progression of Hepatocellular Carcinoma. Environ Toxicol (2022). doi: 10.1002/tox.23489 35179300

[113] Li L Li C Wang S Wang Z Jiang J Wang W. Exosomes Derived From Hypoxic Oral Squamous Cell Carcinoma Cells Deliver miR-21 to Normoxic Cells to Elicit a Prometastatic Phenotype. Cancer Res (2016) 76:1770–80. doi: 10.1158/0008-5472.CAN-15-1625 26992424

[114] Li L Cao B Liang X Lu S Luo H Wang Z. Microenvironmental Oxygen Pressure Orchestrates an Anti- and Pro-Tumoral Gammadelta T Cell Equilibrium *via* Tumor-Derived Exosomes. Oncogene (2019) 38:2830–43. doi: 10.1038/s41388-018-0627-z 30546089

[115] Mao Y Wang Y Dong L Zhang Y Zhang Y Wang C. Hypoxic Exosomes Facilitate Angiogenesis and Metastasis in Esophageal Squamous Cell Carcinoma Through Altering the Phenotype and Transcriptome of Endothelial Cells. J Exp Clin Cancer Res (2019) 38:389. doi: 10.1186/s13046-019-1384-8 31488217 PMC6727585

[116] Xia X Wang S Ni B Xing S Cao H Zhang Z. Hypoxic Gastric Cancer-Derived Exosomes Promote Progression and Metastasis *via* MiR-301a-3p/PHD3/HIF-1alpha Positive Feedback Loop. Oncogene (2020) 39:6231–44. doi: 10.1038/s41388-020-01425-6 32826951

[117] Wang Y Cen A Yang Y Ye H Li J Liu S. miR-181a, Delivered by Hypoxic PTC-Secreted Exosomes, Inhibits DACT2 by Downregulating MLL3, Leading to YAP-VEGF-Mediated Angiogenesis. Mol Ther Nucleic Acids (2021) 24:610–21. doi: 10.1016/j.omtn.2021.02.027 PMC8054101 33898109

[118] Park JE Tan HS Datta A Lai RC Zhang H Meng W. Hypoxic Tumor Cell Modulates its Microenvironment to Enhance Angiogenic and Metastatic Potential by Secretion of Proteins and Exosomes. Mol Cell Proteomics (2010) 9:1085–99. doi: 10.1074/mcp.M900381-MCP200 PMC2877972 20124223

[119] Wozniak M Peczek L Czernek L Duchler M. Analysis of the miRNA Profiles of Melanoma Exosomes Derived Under Normoxic and Hypoxic Culture Conditions. Anticancer Res (2017) 37:6779–89. doi: 10.21873/anticanres.12138 29187456

[120] Khalyfa A Trzepizur W Gileles-Hillel A Qiao Z Sanz-Rubio D Marin JM. Heterogeneity of Melanoma Cell Responses to Sleep Apnea-Derived Plasma Exosomes and to Intermittent Hypoxia. Cancers (Basel) (2021) 13:4781. doi: 10.3390/cancers13194781 34638272 PMC8508428

[121] Park JE Dutta B Tse SW Gupta N Tan CF Low JK. Hypoxia-Induced Tumor Exosomes Promote M2-Like Macrophage Polarization of Infiltrating Myeloid Cells and microRNA-Mediated Metabolic Shift. Oncogene (2019) 38:5158–73. doi: 10.1038/s41388-019-0782-x 30872795

[122] de Jong OG Verhaar MC Chen Y Vader P Gremmels H Posthuma G. Cellular Stress Conditions are Reflected in the Protein and RNA Content of Endothelial Cell-Derived Exosomes. J Extracell Vesicles (2012) 1:10.3402. doi: 10.3402/jev.v1i0.18396 PMC3760650 24009886

[123] Kling MJ Chaturvedi NK Kesherwani V Coulter DW McGuire TR Sharp JG. Exosomes Secreted Under Hypoxia Enhance Stemness in Ewing’s Sarcoma Through miR-210 Delivery. Oncotarget (2020) 11:3633–45. doi: 10.18632/oncotarget.27702 PMC7546758 33088424

[124] Lee JY Ryu D Lim SW Ryu KJ Choi ME Yoon SE. Exosomal miR-1305 in the Oncogenic Activity of Hypoxic Multiple Myeloma Cells: A Biomarker for Predicting Prognosis. J Cancer (2021) 12:2825–34. doi: 10.7150/jca.55553 PMC8040895 33854583

[125] Umezu T Tadokoro H Azuma K Yoshizawa S Ohyashiki K Ohyashiki JH. Exosomal miR-135b Shed From Hypoxic Multiple Myeloma Cells Enhances Angiogenesis by Targeting Factor-Inhibiting HIF-1. Blood (2014) 124:3748–57. doi: 10.1182/blood-2014-05-576116 PMC4263983 25320245

[126] Zhang J Ma J Long K Qiu W Wang Y Hu Z. Overexpression of Exosomal Cardioprotective miRNAs Mitigates Hypoxia-Induced H9c2 Cells Apoptosis. Int J Mol Sci (2017) 18:711. doi: 10.3390/ijms18040711 PMC5412297 28350318

[127] Zhu J Lu K Zhang N Zhao Y Ma Q Shen J. Myocardial Reparative Functions of Exosomes From Mesenchymal Stem Cells are Enhanced by Hypoxia Treatment of the Cells via Transferring microRNA-210 in an Nsmase2-Dependent Way. Artif Cells Nanomed Biotechnol (2018) 46:1659–70. doi: 10.1080/21691401.2017.1388249 PMC5955787 29141446

[128] Liu W Zhang H Mai J Chen Z Huang T Wang S. Distinct Anti-Fibrotic Effects of Exosomes Derived From Endothelial Colony-Forming Cells Cultured Under Normoxia and Hypoxia. Med Sci Monit (2018) 24:6187–99. doi: 10.12659/MSM.911306 PMC6134891 30183690

[129] Khalyfa A Zhang C Khalyfa AA Foster GE Beaudin AE Andrade J. Effect on Intermittent Hypoxia on Plasma Exosomal Micro RNA Signature and Endothelial Function in Healthy Adults. Sleep (2016) 39:2077–90. doi: 10.5665/sleep.6302 PMC5103796 27634792

[130] Sun L Zhu W Zhao P Wang Q Fan B Zhu Y. Long Noncoding RNA UCA1 From Hypoxia-Conditioned hMSC-Derived Exosomes: A Novel Molecular Target for Cardioprotection Through miR-873-5p/XIAP Axis. Cell Death Dis (2020) 11:696. doi: 10.1038/s41419-020-02783-5 32826854 PMC7442657

[131] Wang Y Zhao R Liu W Wang Z Rong J Long X. Exosomal CircHIPK3 Released From Hypoxia-Pretreated Cardiomyocytes Regulates Oxidative Damage in Cardiac Microvascular Endothelial Cells *via* the miR-29a/IGF-1 Pathway. Oxid Med Cell Longev (2019) 2019:7954657. doi: 10.1155/2019/7954657 31885817 PMC6915129

[132] Wang Y Zhao R Shen C Liu W Yuan J Li C. Exosomal CircHIPK3 Released From Hypoxia-Induced Cardiomyocytes Regulates Cardiac Angiogenesis After Myocardial Infarction. Oxid Med Cell Longev (2020) 2020:8418407. doi: 10.1155/2020/8418407 32733638 PMC7376438

[133] Yang Y Li Y Chen X Cheng X Liao Y Yu X. Exosomal Transfer of miR-30a Between Cardiomyocytes Regulates Autophagy After Hypoxia. J Mol Med (Berl) (2016) 94:711–24. doi: 10.1007/s00109-016-1387-2 26857375

[134] Wang L Zhang J. Exosomal lncRNA AK139128 Derived From Hypoxic Cardiomyocytes Promotes Apoptosis and Inhibits Cell Proliferation in Cardiac Fibroblasts. Int J Nanomedicine (2020) 15:3363–76. doi: 10.2147/IJN.S240660 PMC7229807 32494135

[135] Zhang Z Xu Y Cao C Wang B Guo J Qin Z. Exosomes as a Messager to Regulate the Crosstalk Between Macrophages and Cardiomyocytes Under Hypoxia Conditions. J Cell Mol Med (2022) 26:1486–500. doi: 10.1111/jcmm.17162 PMC8899199 35088943

[136] Lin B Chen X Lu C Xu J Qiu Y Liu X. Loss of Exosomal lncRNA HCG15 Prevents Acute Myocardial Ischemic Injury Through the NF-Kappab/P65 and P38 Pathways. Cell Death Dis (2021) 12:1007. doi: 10.1038/s41419-021-04281-8 34707098 PMC8551195

[137] Bian S Zhang L Duan L Wang X Min Y Yu H. Extracellular Vesicles Derived From Human Bone Marrow Mesenchymal Stem Cells Promote Angiogenesis in a Rat Myocardial Infarction Model. J Mol Med (Berl) (2014) 92:387–97. doi: 10.1007/s00109-013-1110-5 24337504

[138] Sano S Izumi Y Yamaguchi T Yamazaki T Tanaka M Shiota M. Lipid Synthesis is Promoted by Hypoxic Adipocyte-Derived Exosomes in 3T3-L1 Cells. Biochem Biophys Res Commun (2014) 445:327–33. doi: 10.1016/j.bbrc.2014.01.183 24513287

[139] Gonzalez-King H Garcia NA Ontoria-Oviedo I Ciria M Montero JA Sepulveda P. Hypoxia Inducible Factor-1alpha Potentiates Jagged 1-Mediated Angiogenesis by Mesenchymal Stem Cell-Derived Exosomes. Stem Cells (2017) 35:1747–59. doi: 10.1002/stem.2618 28376567

[140] Gao W He R Ren J Zhang W Wang K Zhu L. Exosomal HMGB1 Derived From Hypoxia-Conditioned Bone Marrow Mesenchymal Stem Cells Increases Angiogenesis *via* the JNK/HIF-1alpha Pathway. FEBS Open Bio (2021) 11:1364–73. doi: 10.1002/2211-5463.13142 PMC8091582 33711197

[141] Xiong Y Tang R Xu J Jiang W Gong Z Zhang L. Sequential Transplantation of Exosomes and Mesenchymal Stem Cells Pretreated With a Combination of Hypoxia and Tongxinluo Efficiently Facilitates Cardiac Repair. Stem Cell Res Ther (2022) 13:63. doi: 10.1186/s13287-022-02736-z 35130979 PMC8822662

[142] Xue C Shen Y Li X Li B Zhao S Gu J. Exosomes Derived From Hypoxia-Treated Human Adipose Mesenchymal Stem Cells Enhance Angiogenesis Through the PKA Signaling Pathway. Stem Cells Dev (2018) 27:456–65. doi: 10.1089/scd.2017.0296 29415626

[143] Namazi H Mohit E Namazi I Rajabi S Samadian A Hajizadeh-Saffar E. Exosomes Secreted by Hypoxic Cardiosphere-Derived Cells Enhance Tube Formation and Increase Pro-Angiogenic miRNA. J Cell Biochem (2018) 119:4150–60. doi: 10.1002/jcb.26621 29243842

[144] Gray WD French KM Ghosh-Choudhary S Maxwell JT Brown ME Platt MO. Identification of Therapeutic Covariant microRNA Clusters in Hypoxia-Treated Cardiac Progenitor Cell Exosomes Using Systems Biology. Circ Res (2015) 116:255–63. doi: 10.1161/CIRCRESAHA.116.304360 PMC4338016 25344555

[145] Dougherty JA Patel N Kumar N Rao SG Angelos MG Singh H. Human Cardiac Progenitor Cells Enhance Exosome Release and Promote Angiogenesis Under Physoxia. Front Cell Dev Biol (2020) 8:130. doi: 10.3389/fcell.2020.00130 32211408 PMC7068154

[146] Ong SG Lee WH Huang M Dey D Kodo K Sanchez-Freire V. Cross Talk of Combined Gene and Cell Therapy in Ischemic Heart Disease: Role of Exosomal microRNA Transfer. Circulation (2014) 130:S60–9. doi: 10.1161/CIRCULATIONAHA.113.007917 PMC4862832 25200057

[147] Cosme J Guo H Hadipour-Lakmehsari S Emili A Gramolini AO. Hypoxia-Induced Changes in the Fibroblast Secretome, Exosome, and Whole-Cell Proteome Using Cultured, Cardiac-Derived Cells Isolated From Neonatal Mice. J Proteome Res (2017) 16:2836–47. doi: 10.1021/acs.jproteome.7b00144 28641008

[148] Han YD Bai Y Yan XL Ren J Zeng Q Li XD. Co-Transplantation of Exosomes Derived From Hypoxia-Preconditioned Adipose Mesenchymal Stem Cells Promotes Neovascularization and Graft Survival in Fat Grafting. Biochem Biophys Res Commun (2018) 497:305–12. doi: 10.1016/j.bbrc.2018.02.076 29428734

[149] Zhang L Wei Q Liu X Zhang T Wang S Zhou L. Exosomal microRNA-98-5p From Hypoxic Bone Marrow Mesenchymal Stem Cells Inhibits Myocardial Ischemia-Reperfusion Injury by Reducing TLR4 and Activating the PI3K/Akt Signaling Pathway. Int Immunopharmacol (2021) 101:107592. doi: 10.1016/j.intimp.2021.107592 34715573

[150] Zhang CS Shao K Liu CW Li CJ Yu BT. Hypoxic Preconditioning BMSCs-Exosomes Inhibit Cardiomyocyte Apoptosis After Acute Myocardial Infarction by Upregulating microRNA-24. Eur Rev Med Pharmacol Sci (2019) 23:6691–9. doi: 10.26355/eurrev_201908_18560 31378912

[151] Cheng H Chang S Xu R Chen L Song X Wu J. Hypoxia-Challenged MSC-Derived Exosomes Deliver miR-210 to Attenuate Post-Infarction Cardiac Apoptosis. Stem Cell Res Ther (2020) 11:224. doi: 10.1186/s13287-020-01737-0 32513270 PMC7278138

[152] Xia W Chen H Xie C Hou M. Long-Noncoding RNA MALAT1 Sponges microRNA-92a-3p to Inhibit Doxorubicin-Induced Cardiac Senescence by Targeting ATG4a. Aging (Albany NY) (2020) 12:8241–60. doi: 10.18632/aging.103136 PMC7244027 32384281

[153] Han Y Ren J Bai Y Pei X Han Y. Exosomes From Hypoxia-Treated Human Adipose-Derived Mesenchymal Stem Cells Enhance Angiogenesis Through VEGF/VEGF-R. Int J Biochem Cell Biol (2019) 109:59–68. doi: 10.1016/j.biocel.2019.01.017 30710751

[154] Zhu LP Tian T Wang JY He JN Chen T Pan M. Hypoxia-Elicited Mesenchymal Stem Cell-Derived Exosomes Facilitates Cardiac Repair Through miR-125b-Mediated Prevention of Cell Death in Myocardial Infarction. Theranostics (2018) 8:6163–77. doi: 10.7150/thno.28021 PMC6299684 30613290

[155] Qi Y Zhu T Zhang T Wang X Li W Chen D. M1 Macrophage-Derived Exosomes Transfer miR-222 to Induce Bone Marrow Mesenchymal Stem Cell Apoptosis. Lab Invest (2021) 101:1318–26. doi: 10.1038/s41374-021-00622-5 34230646

[156] Namazi H Namazi I Ghiasi P Ansari H Rajabi S Hajizadeh-Saffar E. Exosomes Secreted by Normoxic and Hypoxic Cardiosphere-Derived Cells Have Anti-Apoptotic Effect. Iran J Pharm Res (2018) 17:377–85. PMC5937107 29755568

[157] Pan T Jia P Chen N Fang Y Liang Y Guo M. Delayed Remote Ischemic Preconditioning ConfersRenoprotection Against Septic Acute Kidney Injury *via* Exosomal miR-21. Theranostics (2019) 9:405–23. doi: 10.7150/thno.29832 PMC6376188 30809283

[158] Guitart K Loers G Buck F Bork U Schachner M Kleene R. Improvement of Neuronal Cell Survival by Astrocyte-Derived Exosomes Under Hypoxic and Ischemic Conditions Depends on Prion Protein. Glia (2016) 64:896–910. doi: 10.1002/glia.22963 26992135

[159] Kang X Jiang L Chen X Wang X Gu S Wang J. Exosomes Derived From Hypoxic Bone Marrow Mesenchymal Stem Cells Rescue OGD-Induced Injury in Neural Cells by Suppressing NLRP3 Inflammasome-Mediated Pyroptosis. Exp Cell Res (2021) 405:112635. doi: 10.1016/j.yexcr.2021.112635 34051241

[160] Cui GH Wu J Mou FF Xie WH Wang FB Wang QL. Exosomes Derived From Hypoxia-Preconditioned Mesenchymal Stromal Cells Ameliorate Cognitive Decline by Rescuing Synaptic Dysfunction and Regulating Inflammatory Responses in APP/PS1 Mice. FASEB J (2018) 32:654–68. doi: 10.1096/fj.201700600R 28970251

[161] Xie JC Ma XY Liu XH Yu J Zhao YC Tan Y. Hypoxia Increases Amyloid-Beta Level in Exosomes by Enhancing the Interaction Between CD147 and Hook1. Am J Transl Res (2018) 10:150–63. PMC5801354 29423001

[162] Xie L Zhao H Wang Y Chen Z. Exosomal Shuttled miR-424-5p From Ischemic Preconditioned Microglia Mediates Cerebral Endothelial Cell Injury Through Negatively Regulation of FGF2/STAT3 Pathway. Exp Neurol (2020) 333:113411. doi: 10.1016/j.expneurol.2020.113411 32707150

[163] Liang Y Wu JH Zhu JH Yang H. Exosomes Secreted by Hypoxia-Pre-Conditioned Adipose-Derived Mesenchymal Stem Cells Reduce Neuronal Apoptosis in Rats With Spinal Cord Injury. J Neurotrauma (2022). doi: 10.1089/neu.2021.0290 35018814

[164] Zhao M Gao Y Wang F Cheng X Zhao T Zhao Y. Neural Progenitor Cells-Secreted Exosomal miR-210 Induced by Hypoxia Influences Cell Viability. Neuroreport (2020) 31:798–805. doi: 10.1097/WNR.0000000000001490 32590394 PMC7340230

[165] Xie J Li X Zhou Y Wu J Tan Y Ma X. Resveratrol Abrogates Hypoxia-Induced Up-Regulation of Exosomal Amyloid-Beta Partially by Inhibiting Cd147. Neurochem Res (2019) 44:1113–26. doi: 10.1007/s11064-019-02742-3 30771155

[166] Xu L Cao H Xie Y Zhang Y Du M Xu X. Exosome-Shuttled miR-92b-3p From Ischemic Preconditioned Astrocytes Protects Neurons Against Oxygen and Glucose Deprivation. Brain Res (2019) 1717:66–73. doi: 10.1016/j.brainres.2019.04.009 30986407

[167] Lee C Mitsialis SA Aslam M Vitali SH Vergadi E Konstantinou G. Exosomes Mediate the Cytoprotective Action of Mesenchymal Stromal Cells on Hypoxia-Induced Pulmonary Hypertension. Circulation (2012) 126:2601–11. doi: 10.1161/CIRCULATIONAHA.112.114173 PMC3979353 23114789

[168] Deng L Blanco FJ Stevens H Lu R Caudrillier A McBride M. MicroRNA-143 Activation Regulates Smooth Muscle and Endothelial Cell Crosstalk in Pulmonary Arterial Hypertension. Circ Res (2015) 117:870–83. doi: 10.1161/CIRCRESAHA.115.306806 PMC4620852 26311719

[169] Zhao L Luo H Li X Li T He J Qi Q. Exosomes Derived From Human Pulmonary Artery Endothelial Cells Shift the Balance Between Proliferation and Apoptosis of Smooth Muscle Cells. Cardiology (2017) 137:43–53. doi: 10.1159/000453544 28068653

[170] Shi XF Wang H Kong FX Xu QQ Xiao FJ Yang YF. Exosomal miR-486 Regulates Hypoxia-Induced Erythroid Differentiation of Erythroleukemia Cells Through Targeting Sirt1. Exp Cell Res (2017) 351:74–81. doi: 10.1016/j.yexcr.2016.12.023 28043832

[171] Tadokoro H Umezu T Ohyashiki K Hirano T Ohyashiki JH. Exosomes Derived From Hypoxic Leukemia Cells Enhance Tube Formation in Endothelial Cells. J Biol Chem (2013) 288:34343–51. doi: 10.1074/jbc.M113.480822 PMC3843049 24133215

[172] Niazi V Ghafouri-Fard S Verdi J Jeibouei S Karami F Pourhadi M. Hypoxia Preconditioned Mesenchymal Stem Cell-Derived Exosomes Induce Ex Vivo Expansion of Umbilical Cord Blood Hematopoietic Stem Cells CD133+ by Stimulation of Notch Signaling Pathway. Biotechnol Prog (2022) 38:e3222. doi:10.1002/btpr.322 34734683 10.1002/btpr.3222

[173] Wang J Wu H Peng Y Zhao Y Qin Y Zhang Y. Hypoxia Adipose Stem Cell-Derived Exosomes Promote High-Quality Healing of Diabetic Wound Involves Activation of PI3K/Akt Pathways. J Nanobiotechnol (2021) 19:202. doi: 10.1186/s12951-021-00942-0 PMC8261989 34233694

[174] Ye L Guo H Wang Y Peng Y Zhang Y Li S. Exosomal Circehmt1 Released From Hypoxia-Pretreated Pericytes Regulates High Glucose-Induced Microvascular Dysfunction *via* the NFIA/NLRP3 Pathway. Oxid Med Cell Longev (2021) 2021:8833098. doi: 10.1155/2021/8833098 33815662 PMC7994074

[175] Zhang XF Wang T Wang ZX Huang KP Zhang YW Wang GL. Hypoxic ucMSC-Secreted Exosomal miR-125b Promotes Endothelial Cell Survival and Migration During Wound Healing by Targeting TP53INP1. Mol Ther Nucleic Acids (2021) 26:347–59. doi: 10.1016/j.omtn.2021.07.014 PMC8416974 34513314

[176] Yu W Zeng H Chen J Fu S Huang Q Xu Y. miR-20a-5p is Enriched in Hypoxia-Derived Tubular Exosomes and Protects Against Acute Tubular Injury. Clin Sci (Lond) (2020) 134:2223–34. doi: 10.1042/CS20200288 32808649

[177] Liang YR Zhang T Jia P Xu XL Fang Y Ding XQ. Interaction Between Bone Marrow-Derived Dendritic Cells and miR-21 of Tubular Renal Epithelial Cells Under Hypoxia. Eur Rev Med Pharmacol Sci (2019) 23:1641–51. doi: 10.26355/eurrev_201902_17124 30840288

[178] Zhou X Zhao S Li W Ruan Y Yuan R Ning J. Tubular Cell-Derived Exosomal miR-150-5p Contributes to Renal Fibrosis Following Unilateral Ischemia-Reperfusion Injury by Activating Fibroblast *In Vitro* and *In Vivo* . Int J Biol Sci (2021) 17:4021–33. doi: 10.7150/ijbs.62478 PMC8495396 34671216

[179] de Jong OG van Balkom BW Gremmels H Verhaar MC. Exosomes From Hypoxic Endothelial Cells Have Increased Collagen Crosslinking Activity Through Up-Regulation of Lysyl Oxidase-Like 2. J Cell Mol Med (2016) 20:342–50. doi: 10.1111/jcmm.12730 PMC4727569 26612622

[180] Salomon C Ryan J Sobrevia L Kobayashi M Ashman K Mitchell M. Exosomal Signaling During Hypoxia Mediates Microvascular Endothelial Cell Migration and Vasculogenesis. PloS One (2013) 8:e68451. doi: 10.1371/journal.pone.0068451 23861904 PMC3704530

[181] Koh YQ Peiris HN Vaswani K Reed S Rice GE Salomon C. Characterization of Exosomal Release in Bovine Endometrial Intercaruncular Stromal Cells. Reprod Biol Endocrinol (2016) 14:78. doi: 10.1186/s12958-016-0207-4 27829441 PMC5103490

[182] Liu W Rong Y Wang J Zhou Z Ge X Ji C. Exosome-Shuttled miR-216a-5p From Hypoxic Preconditioned Mesenchymal Stem Cells Repair Traumatic Spinal Cord Injury by Shifting Microglial M1/M2 Polarization. J Neuroinflamm (2020) 17:47. doi: 10.1186/s12974-020-1726-7 PMC7001326 32019561

[183] Liu W Li L Rong Y Qian D Chen J Zhou Z. Hypoxic Mesenchymal Stem Cell-Derived Exosomes Promote Bone Fracture Healing by the Transfer of miR-126. Acta Biomater (2020) 103:196–212. doi: 10.1016/j.actbio.2019.12.020 31857259

[184] Thankam FG Chandra I Diaz C Dilisio MF Fleegel J Gross RM. Matrix Regeneration Proteins in the Hypoxia-Triggered Exosomes of Shoulder Tenocytes and Adipose-Derived Mesenchymal Stem Cells. Mol Cell Biochem (2020) 465:75–87. doi: 10.1007/s11010-019-03669-7 31797254 PMC6957752

[185] Chu X Chen L Zhang Y. Exosomes Derived From PMN-MDSCs Preconditioned by Hypoxia Attenuate Arthropathy of Collagen-Induced Arthritis Mice. Xi Bao Yu Fen Zi Mian Yi Xue Za Zhi (2021) 37:728–35. 34236033

[186] Yuan N Ge Z Ji W Li J. Exosomes Secreted From Hypoxia-Preconditioned Mesenchymal Stem Cells Prevent Steroid-Induced Osteonecrosis of the Femoral Head by Promoting Angiogenesis in Rats. BioMed Res Int (2021) 2021:6655225. doi: 10.1155/2021/6655225 33928159 PMC8049797

[187] Ding Y Wang L Wu H Zhao Q Wu S. Exosomes Derived From Synovial Fibroblasts Under Hypoxia Aggravate Rheumatoid Arthritis by Regulating Treg/Th17 Balance. Exp Biol Med (Maywood) (2020) 245:1177–86. doi: 10.1177/1535370220934736 PMC7437376 32615822

[188] Mayo JN Bearden SE. Driving the Hypoxia-Inducible Pathway in Human Pericytes Promotes Vascular Density in an Exosome-Dependent Manner. Microcirculation (2015) 22:711–23. doi: 10.1111/micc.12227 PMC4715585 26243428

[189] Shao M Jin M Xu S Zheng C Zhu W Ma X. Exosomes From Long Noncoding RNA-Gm37494-ADSCs Repair Spinal Cord Injury *via* Shifting Microglial M1/M2 Polarization. Inflammation (2020) 43:1536–47. doi: 10.1007/s10753-020-01230-z 32307615

[190] Yoon JH Kim J Kim KL Kim DH Jung SJ Lee H. Proteomic Analysis of Hypoxia-Induced U373MG Glioma Secretome Reveals Novel Hypoxia-Dependent Migration Factors. Proteomics (2014) 14:1494–502. doi: 10.1002/pmic.201300554 24729417

[191] Jung KO Jo H Yu JH Gambhir SS Pratx G. Development and MPI Tracking of Novel Hypoxia-Targeted Theranostic Exosomes. Biomaterials (2018) 177:139–48. doi: 10.1016/j.biomaterials.2018.05.048 PMC6019194 29890363

[192] Saravanan PB Vasu S Yoshimatsu G Darden CM Wang X Gu J. Differential Expression and Release of Exosomal miRNAs by Human Islets Under Inflammatory and Hypoxic Stress. Diabetologia (2019) 62:1901–14. doi: 10.1007/s00125-019-4950-x 31372667

[193] Agarwal U George A Bhutani S Ghosh-Choudhary S Maxwell JT Brown ME. Experimental, Systems, and Computational Approaches to Understanding the MicroRNA-Mediated Reparative Potential of Cardiac Progenitor Cell-Derived Exosomes From Pediatric Patients. Circ Res (2017) 120:701–12. doi: 10.1161/CIRCRESAHA.116.309935 PMC5315680 27872050

[194] Zhang W Zhou X Yao Q Liu Y Zhang H Dong Z. HIF-1-Mediated Production of Exosomes During Hypoxia is Protective in Renal Tubular Cells. Am J Physiol Renal Physiol (2017) 313:F906–13. doi: 10.1152/ajprenal.00178.2017 PMC5668579 28679592

[195] Biro O Alasztics B Molvarec A Joo J Nagy B Rigo JJ. Various Levels of Circulating Exosomal Total-miRNA and miR-210 hypoxamiR in Different Forms of Pregnancy Hypertension. Pregnancy Hypertens (2017) 10:207–12. doi: 10.1016/j.preghy.2017.09.002 29153681

[196] Yang W Ma J Zhou W Cao B Zhou X Zhang H. Reciprocal Regulations Between miRNAs and HIF-1alpha in Human Cancers. Cell Mol Life Sci (2019) 76:453–71. doi: 10.1007/s00018-018-2941-6 PMC11105242 30317527

[197] Datta A Kim H McGee L Johnson AE Talwar S Marugan J. High-Throughput Screening Identified Selective Inhibitors of Exosome Biogenesis and Secretion: A Drug Repurposing Strategy for Advanced Cancer. Sci Rep (2018) 8:8161. doi: 10.1038/s41598-018-26411-7 29802284 PMC5970137

[198] Catalano M O’Driscoll L. Inhibiting Extracellular Vesicles Formation and Release: A Review of EV Inhibitors. J Extracell Vesicles (2020) 9:1703244. doi: 10.1080/20013078.2019.1703244 32002167 PMC6968539

[199] Zhang H Lu J Liu J Zhang G Lu A. Advances in the Discovery of Exosome Inhibitors in Cancer. J Enzyme Inhib Med Chem (2020) 35:1322–30. doi: 10.1080/14756366.2020.1754814 PMC7717571 32543905

[200] Hao S Liu Y Yuan J Zhang X He T Wu X. Novel Exosome-Targeted CD4+ T Cell Vaccine Counteracting CD4+25+ Regulatory T Cell-Mediated Immune Suppression and Stimulating Efficient Central Memory CD8+ CTL Responses. J Immunol (2007) 179:2731–40. doi: 10.4049/jimmunol.179.5.2731 PMC2567870 17709486

[201] Dai S Wei D Wu Z Zhou X Wei X Huang H. Phase I Clinical Trial of Autologous Ascites-Derived Exosomes Combined With GM-CSF for Colorectal Cancer. Mol Ther (2008) 16:782–90. doi: 10.1038/mt.2008.1 PMC7106337 18362931

[202] Brinton LT Sloane HS Kester M Kelly KA. Formation and Role of Exosomes in Cancer. Cell Mol Life Sci (2015) 72:659–71. doi: 10.1007/s00018-014-1764-3 PMC5489338 25336151

[203] Natasha G Gundogan B Tan A Farhatnia Y Wu W Rajadas J. Exosomes as Immunotheranostic Nanoparticles. Clin Ther (2014) 36:820–9. doi: 10.1016/j.clinthera.2014.04.019 24863261

